# Dual Inhibition of COX‐2/5‐LOX Through Novel Hybrids of NSAIDs and Peptides: Insights from Molecular Dynamics Simulation and Per‐Residue Decomposition

**DOI:** 10.1002/open.202500452

**Published:** 2026-03-20

**Authors:** J. Carlos Jiménez‐Cruz, Ramón Guzmán‐Mejía, Pedro Navarro‐Santos, Hugo A. García‐Gutiérrez, Julio C. Ontiveros‐Rodríguez, Rafael Herrera‐Bucio, Verónica Cortés‐Muñoz, Judit A. Aviña‐Verduzco

**Affiliations:** ^1^ Secretaría de Ciencia, Humanidades, Tecnología e Innovación‐Instituto de Investigaciones Químico Biológicas Universidad Michoacana de San Nicolás de Hidalgo Morelia Michoacán Mexico; ^2^ Instituto de Investigaciones Químico Biológicas Universidad Michoacana de San Nicolás de Hidalgo Morelia Michoacán México

**Keywords:** amino acids, COX‐2/5‐LOX, hybrid, MMPBSA, NSAID

## Abstract

The present study describes the synthesis of an analog of the anti‐inflammatory drug fenbufen, a compound which has recently attracted of renewed interest in the treatment of chronic degenerative diseases such as cancer and rheumatoid arthritis. The molecule under consideration, 4‐(4‐methoxyphenyl)‐4‐oxobutanoic acid, is distinguished from fenbufen by the absence of the biphenyl moiety. This characteristic results in the generation of nontoxic metabolites during biotransformation. The incorporation of aromatic amino acid residues (phenylalanine and tyrosine) led to the modulation of the hybrid NSAID–amino acid and NSAID–peptide derivatives, thereby altering their biological activity and safety profile. The inhibitory activity against the dual cyclooxygenase‐2/5‐lipoxygenase system was evaluated using molecular docking, free energy calculations, and per‐residue decomposition analysis based on molecular dynamics simulations. Compounds containing a tyrosine residue or the Tyr‐Phe dipeptide exhibited strong drug–receptor binding affinities and long‐term stability during our simulations, in some cases outperforming the reference drugs celecoxib and zileuton. The values of the free energy (Δ*G*) obtained through the Boltzmann equation demonstrated a strong correlation with the energy decomposition data, particularly for the lead compounds, which exhibited stabilizing interactions with the heme prosthetic group and the Fe^2+^ ion. These findings provide substantial support for the identification of these molecules as promising candidates for further preclinical development.

## Graphical abstract

Hybrid NSAID–aminoacid molecules have demonstrated excellent activity in inhibiting the cyclooxygenase/lipoxygenase system. Accordingly, the present study describes the synthesis of molecules of this kind, examining their behavior via docking, molecular dynamics simulations and per‐residue energy decomposition analyses. The molecule with the tyrosine residue interacted better than zileuton and celecoxib, which were used as standards. Consequently, it will be chosen for preclinical testing.

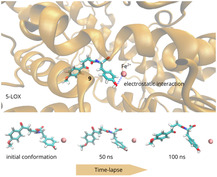

## 1 Introduction

Acute inflammation is defined as a short‐term defensive response that is triggered by harmful stimuli, including infection, trauma, or exposure to physical, chemical, or allergenic agents [1].

From a biochemical perspective, the initiation of the response leads to the production of proinflammatory cytokines including interleukins (IL‐1, IL‐2, IL‐6, IL‐7) and tumor necrosis factor‐alpha (TNF‐α) [2, 3]. These regulate gene transcription and play a direct role in the activation and differentiation of innate immune cells such as mast cells and tissue‐resident macrophages. These cells further influence the balance between pro‐ and anti‐inflammatory signaling through the modulation of additional cytokines [4].

The sustained elevation of proinflammatory cytokines, whether due to infection or autoimmune processes can result in a condition known as a cytokine storm [5], which has been implicated in the high mortality associated with diseases such as severe acute respiratory syndrome (SARS)‐CoV‐2‐induced pneumonia or COVID‐19 [6]. Notably, in addition to the overexpression of IL‐6 [7], the viral spike protein is capable of activating the cyclooxygenase‐2 (COX‐2) isoenzyme [8], thus exacerbating the inflammatory response and contributing to more severe disease progression [9]. Additionally, the overactivation of 5‐lipoxygenase (5‐LOX), which converts arachidonic acid into proinflammatory leukotrienes (LTs), further promotes the release of cytokines such as TNF‐α, IL‐1α and IL‐1β [10].

Consequently, the dual inhibition of COX‐2 and 5‐LOX has emerged as a promising therapeutic strategy not only for emerging infectious diseases like COVID‐19 [11], but also for chronic inflammatory conditions, including various forms of arthritis [12].

One effective approach to achieve COX‐2 selectivity entails the conjugation of classical NSAID scaffolds with amino acids [13, 14], or peptides [15]. Hybrid molecules of this nature are generally larger and better suited to interact with the broader COX‐2 active site, while being sterically excluded from COX‐1, whose binding pocket is approximately 25% smaller due to residue substitutions such as Ala523 (COX‐2) being replaced by Ile523 in COX‐1 [16].

Short peptides have also demonstrated the capacity of inhibit COX‐2 with high efficiency, with certain tripeptides achieving activity levels comparable to celecoxib (Cel) [17]. Moreover, co‐administration of NSAIDs with wheat‐derived peptides has demonstrated a protective effect against gastrointestinal damage and reduced TNF‐α levels [18]. Recent investigations have explored the use of ionic NSAID‐amino acid salts (NSAID‐aa) [19], as well as free amino acids, to control inflammation and inhibit SARS‐CoV‐2 replication [20, 21], through various modalities including conjugation, linker‐based design [22], with covalent bonding [15]. In this context, a series of naproxen (Npx)‐dehydrodipeptide hybrids were developed using amino acids as central linkers and *O*‐terminal residues such as dehydrophenylalanine (ΔPhe) and dehydroaminobutyrate (ΔAbu) [23]. Among these, Npx‐*L*‐Z‐Δphe‐OH emerged as a lead compound, exhibiting comparable bioactivity to naproxen and significant 5‐LOX inhibition at 100 μM. Selectivity was further enhanced by incorporating aromatic *D*‐amino acids into the Npx framework, achieving selectivity ratios of up to 20:1 compared with commercial NSAIDs [13, 14].

Building upon this facts, the same research group examined additional NSAID‐peptide conjugates involving (*R*)‐furbiprofen, (*R*,*S*)‐furbiprofen, (*R*,*S*)‐ibuprofen and aspirin. These hybrids conjugated with aromatic peptides exhibited strong COX inhibition and also formed supramolecular gels with potential for additional therapeutic applications [14]. In addition, Npx was successfully conjugated to free amino acids and methyl amino esters via thiourea‐type linkers, producing hydrophobic compounds that enhanced interactions with key residues in the enzyme's binding pocket [24].

Recent studies have reported that diclofenac hybrids with proline or tyrosine, as well as with trolox and *p*‐coumaric acid analogs, exhibit anti‐inflammatory potency up to 2.5 times greater than diclofenac alone [25]. Additionally, the natural amino acid‐derived compounds tyrosol and tryptophol derived from Tyr and Trp, respectively have demonstrated significant anti‐inflammatory activity [26, 27].

## 2 Results and Discussion

The synthetic route toward the development of novel NSAID‐aa and NSAID‐peptide hybrids was initiated through with the one‐step preparation of 4‐(4‐methoxyphenyl)‐4‐oxobutanoic acid (**1**), a structural analog of the anti‐inflammatory agent fenbufen (4‐oxo‐4‐(4‐phenylphenyl)butanoic acid). This outcome was accomplished through the utilization of a Heck coupling between 2,3‐dihydrofuran (2,3‐DHF) and 4‐iodoanisole, followed by heterocyclic ring opening under microwave irradiation in the presence of the *trans*‐PdCl_2_Gly_2_ catalyst [28]. The reaction efficiently afforded ketol intermediate **1**. It is important to note that under conventional reflux conditions (80–90°C), the reaction predominantly affords the Heck product **1a**, while 2,3‐DHF ring opening is disfavored [29].

The ketol intermediate **1** was then subjected to Corey–Schmidt oxidation, furnishing carboxylic acid **3** in good yield. The acid **3** was subsequently activated via mixed anhydride methodology and coupled to the amino group of pre‐esterified *L*‐phenylalanine **4** and *L*‐tyrosine **5**, resulting in the single‐residue conjugates **6** and **7**.

**SCHEME 1:**
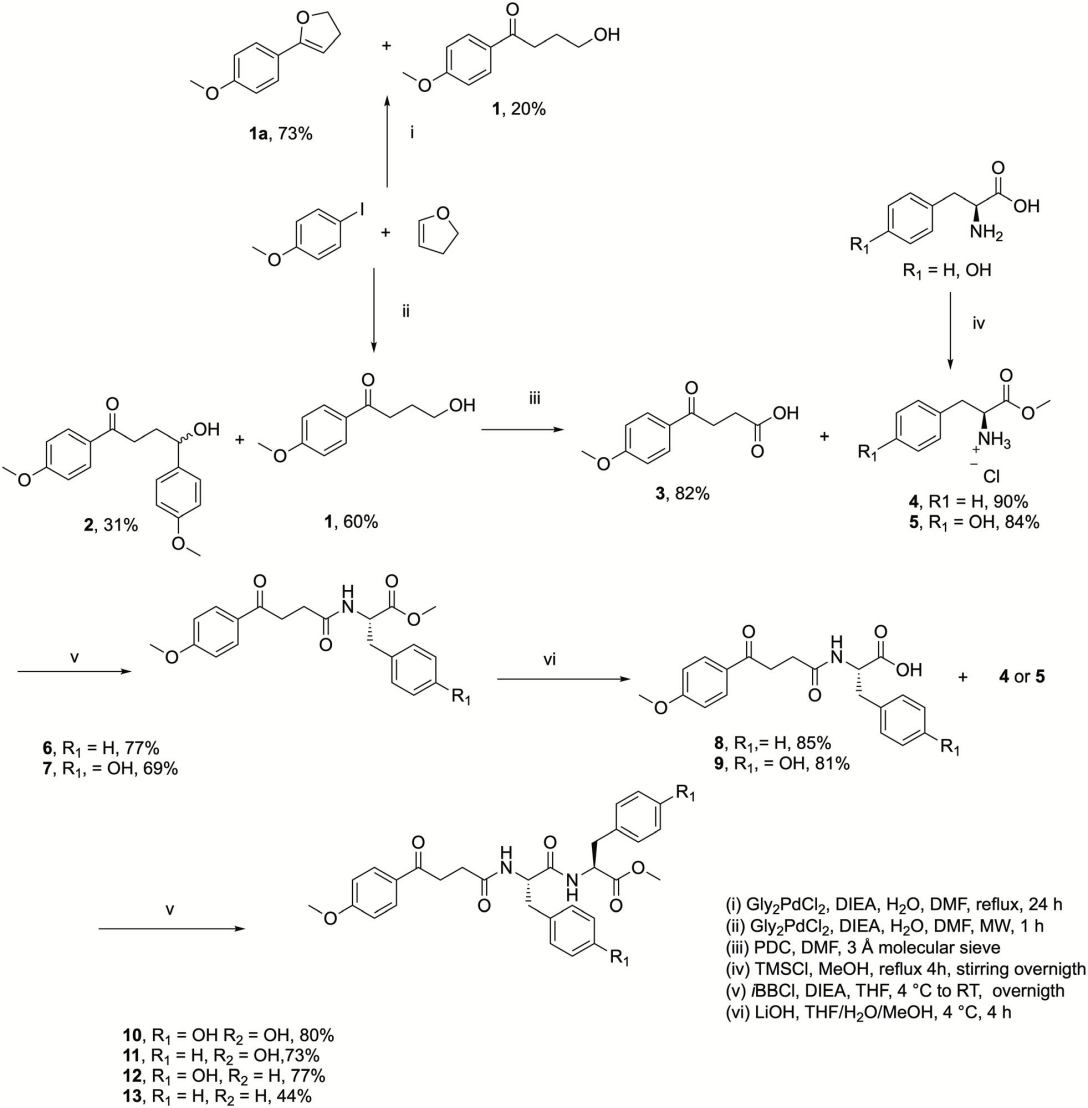
Synthetic route for the preparation of NSAID‐peptide hybrid compounds.

To construct NSAID‐peptide hybrids, compounds **6** and **7** were hydrolyzed using LiOH to yield carboxylic acids **8** and **9**. These intermediates were further coupled to a second equivalent of *L*‐phenylalanine or *L*‐tyrosine to generate the corresponding dipeptide hybrids: methyl (4‐(4‐methoxyphenyl)‐4‐oxobutanoyl)‐*L*‐tyrosyl‐*L*‐tyrosinate (**10**), methyl (4‐(4‐methoxyphenyl)‐4‐oxobutanoyl)‐*L*‐phenylalanyl‐*L*‐tyrosinate (**11**), methyl (4‐(4‐methoxyphenyl)‐4‐oxobutanoyl)‐*L*‐tyrosyl‐*L*‐phenylalaninate (**12**) and methyl (4‐(4‐methoxyphenyl)‐4‐oxobutanoyl)‐*L*‐phenylalanyl‐*L*‐phenylalaninate (**13**). In all four cases, **10** **–13**, the *C*‐terminal residue was maintained as a methyl ester, a strategy aimed at reducing overall polarity and enhancing both solubility and transdermal absorption. These methyl ester derivatives demonstrated good solubility in acetone, water and DMSO. Interestingly, upon dissolution in polar solvents such as DMSO, some hybrids exhibited supramolecular gel formation, suggesting additional potential for formulation‐based applications (Scheme 1).

### 2.1 Molecular Docking

The oxidoreductase enzyme COX‐2 (PDB ID: 3LN1) [30], which contains cocrystallized celecoxib, was selected as the target protein for molecular docking. Celecoxib is a time‐dependent COX‐2 inhibitor with slow reversible binding [31] and its high potency and isoform selectivity make it a suitable reference for the evaluation of newly synthesized analogs [16].

To validate the docking protocol, a redocking experiment was performed using the native ligand. The aim is to ensure accurate reproduction of the crystallographic pose within the catalytic site, as assessed by the root‐mean‐square deviation (RMSD). An RMSD value of 0.99 Å was calculated, thereby confirming the reliability of the docking approach and its ability to replicate the binding geometry reported in the crystal structure.

The predicted binding pose of celecoxib shown a retention of three of the four hydrogen bonds that were observed in the original COX‐2‐celecoxib crystal structure, specifically with residues Gln178, Arg499 and Phe504. Additional hydrophobic interactions were identified with Val335, Leu370 and Ala513. The binding affinity calculated for the celecoxib‐3LN1 complex was –11.01 kcal/mol, serving as the reference value for subsequent comparison.

Docking simulations were then conducted for compounds **8** through **13** (shown in Table 1, column 3LN1). Among them, ligands **8**, **11** and **13** exhibited favorable binding affinities (–10.38, –11.10, and –10.57 kcal/mol, respectively) and established key interactions, including hydrogen bonds with residues such as His75, Tyr371, Arg499 and networks of hydrophobic contacts. However, despite their thermodynamic favorability, these ligands failed to align their pharmacophores consistently within the COX‐2 binding pocket relative to celecoxib, particularly when considering the spatial distribution of hydrophilic and hydrophobic regions.

**TABLE 1:** Binding affinities (kcal/mol) of ligands **8**–**13** against the COX‐2 and 5‐LOX enzymes (PDB:3LN1, 3O8Y) and the main residue interactions within the binding pocket.

Entry	Compound	Binding free energy, kcal/mol		Residues in binding pockets (3LN1, COX‐2)	Residues in binding pockets (3O8Y, 5‐LOX)
		3LN1 (COX‐2)	3O8Y (5‐LOX)		
**1**	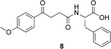	−10.38	−9.06	Hydrogen bond: His75, Tyr371, Arg499; π–π T‐shaped: Phe504; Alkyl: Leu370; π‐alkyl: His75, Leu338, Tyr371, Trp373, Leu370, Val509	Hydrogen bond: Ala424, His367; π–π T‐shaped: Phe421; Alkyl: Lys423, Ala424; π‐alkyl: Ala410, Leu414, Leu368, Trp559, Ala603
**2**	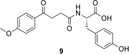	−9.89	−9.05	Hydrogen bond: Gln178, Tyr341, Tyr371, Phe504; Carbon hydrogen bond: Ser339; Amide‐π shaped: Trp373; Alkyl: Arg499, Ala502, Val509; π‐σ: Val509; Alkyl, π‐alkyl: Val330, Tyr334, Val335, Ala502	Hydrogen bond: Gln363, Thr364, Ala424, Ile673; π‐donor hydrogen: Asn425; π–π T‐shaped: Phe421; Alkyl: Lys423; π‐alkyl Leu368, Ala424, Ala410, Leu414, Trp599, Ala603
**3**	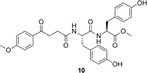	−10.09	−6.89	Van der Waals: Ser339; Hydrogen bond: Ile503, Phe504; Carbon hydrogen bond: Ala513; π‐cation: Arg106 Leu517	Hydrogen bond: Tyr181, Gln363, Thr364, Asn425; amide‐π stacked: Trp599; Alkyl: Leu368, Leu373, Ala410, Arg411, Ala603, Leu607; π‐alkyl: Leu368, Ala424, Ala410, Leu414, Trp599, Ala603; π‐σ: Ile415, Ala603; π‐alkyl: Leu368, Phe359, Ala410, Ala424
**4**	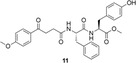	−11.10	−9.2	Hydrogen bond: Tyr371; Carbon hydrogen bond: Ser339; π–π T‐shaped: Val102, His75, Tyr341, Phe504; π‐σ:. Val509; Alkyl: Val330, Val335, leu517, Leu520; π‐alkyl: Leu78, Val102, Tyr334, Val335, Leu345, Arg499, Ala502	Hydrogen bond: Asn425, Ala424, His367; Amide‐π stacked: Ala410; Alkyl: Leu368, Leu373, Arg411, Ile415; π‐alkyl: Leu368, Phe369, Ala410, Leu414, Ile415, Ala424, Lys423, His600, Ala603
**5**	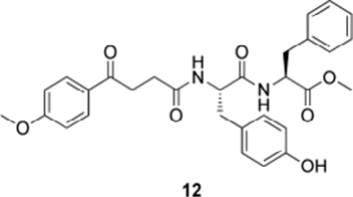	−11.01	−7.3	Hydrogen bond: Gln178, Leu338, Tyr371, Phe504; π–π T‐shaped: Tyr341, Tyr371; Alkyl: Val102, Val330, Val335, Leu517; π‐σ: Val102, Val509; π‐alkyl: Leu78, Leu338	Hydrogen bond: Gln373, His367, His372, (no classical) His600, Asn407; Electrostacic π‐anion: Ile673; π–π T‐shaped: His372; Alkyl: Leu368, Ala410, Ala603, Val604; π‐σ: Ile415, Leu607, Trp509; π‐alkyl: Phe359, His372, Ala410, Leu414, Ile415, Lys423, Ala424, His600, Ala603, Val604
**6**	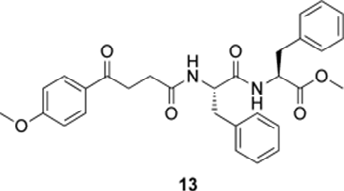	**−**10.57	−8.90	Van der Waals: Ala513; Hydrogen bond: Arg106, Tyr341; π‐σ: Leu78, Val102, Ser339, Val509; π‐sulfur: Met508; π–π T‐shaped: Tyr371; π–π stacked: Gly512, Leu338; Alkyl, π‐alkyl: Leu78, Ile98, Val102, Tyr101	Hydrogen bond: Tyr181, Gln363, His367; Amide‐π stacked: His372; Alkyl: Arg411, Leu373, Leu368, Ala410, Ile415, Ala424, Pro569; Alkyl: Leu368, Ala424, Ala410, Leu414, Trp599, Ala603; π‐alkyl: Ile406, Ala410, Leu368, Leu414, Ile415, Phe359, Lys423, Ala424, Ala603, His600

**FIGURE 1:**
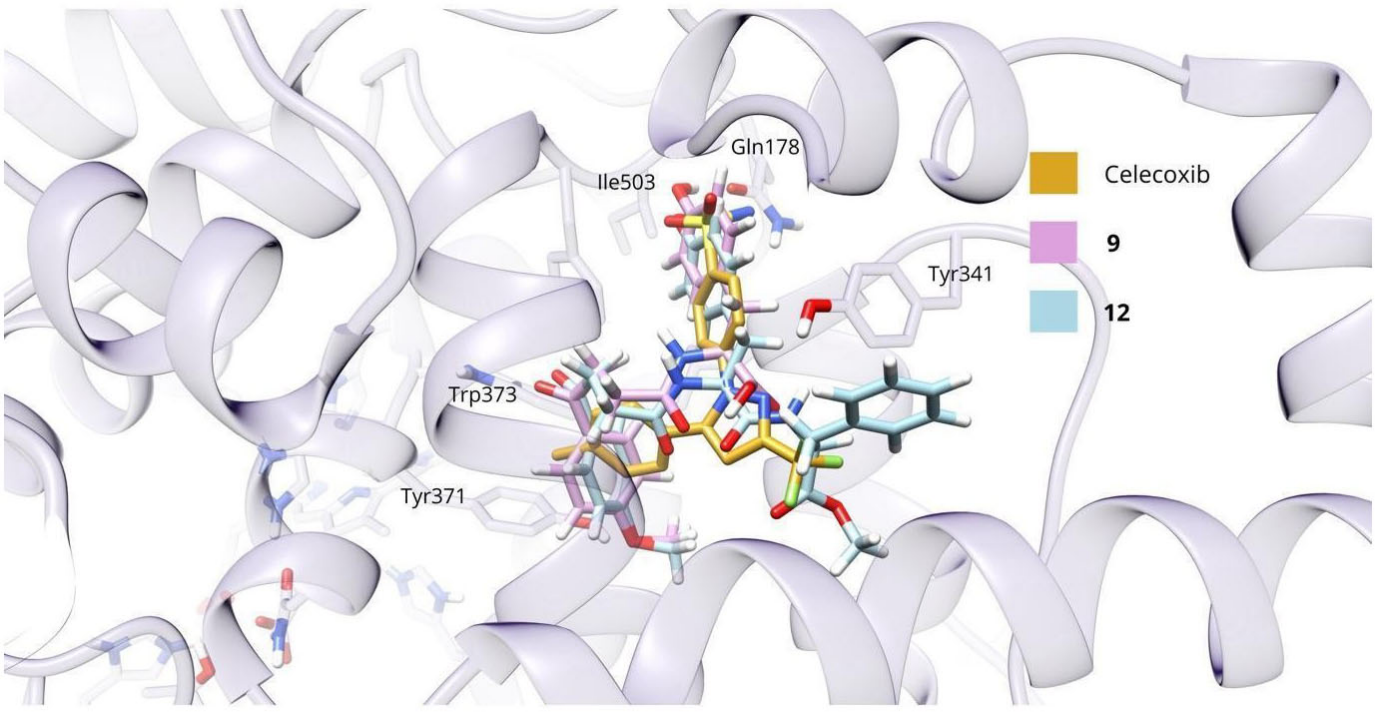
Pose adopted within the COX‐2 catalytic site by ligands **9** and **12** versus celecoxib.

However, ligands **9**, **10** and **12** exhibited more favorable conformational characteristics. These compounds adopted an extended conformation involving the 4‐methoxyphenyl‐3‐propanoylcarboxamide moiety and the tyrosine residue, linked via an amide bond an arrangement reminiscent of the binding mode observed for arachidonic acid, the enzyme's natural substrate [31]. Notably, these ligands occupied similar subpockets within the active site as celecoxib. The anisole group was positioned within the same hydrophilic region targeted by the reference drug's methylbenzene moiety (Figure 1), while the tyrosine's 4‐hydroxyl group extended into the polar zone where celecoxib places its sulfonamide, a key hydrogen‐bonding region. Additionally, the amide functionalities of the ligands were oriented near the position of the pyrazole ring's amino groups for the case of celecoxib, further reinforcing mimicry of its interaction profile. In terms of binding energy, ligands **9**, **10** and **12** exhibited values of −9.89, −10.09 and –11.01 kcal/mol, respectively, the latter matching the reference binding energy of celecoxib. These findings suggested that spatial alignment and interaction mimicry, rather than affinity alone, are critical for achieving effective COX‐2 inhibition, thereby ligands **9** and **12** were considered as particularly compelling candidates for further biological evaluation.

Furthermore, the interaction profiles of these ligands highlight structurally relevant features within the COX‐2 active site. For ligand **9**, a well‐developed hydrogen‐bonding network is observed within the polar region of the catalytic pocket. The tyrosine hydroxyl group of the ligand forms hydrogen bonds with Gln178 and Phe504, while the carboxylate moiety establishes two hydrogen bonds with Tyr341. Furthermore, Tyr371 interacts via hydrogen bonding with the methoxy oxygen of the anisole fragment.

Ligand **12**, regarded as one of the most promising candidates, adopts an extended conformation that spans the entire binding cavity due to the presence of three aromatic rings. This ligand forms four hydrogen bonds: three involving the tyrosine –OH group and residues Gln178, Phe504 and Leu338, as well as an additional interaction with Tyr371.

In contrast, ligand **13** assumes a folded conformation similar to that of ligand **12** and engages in two hydrogen bonds between the tyrosine hydroxyl group and residues Ile503 and Phe504.

These hydrogen‐bonding interactions are of particular relevance, as they facilitate in the binding of selective inhibitors such as celecoxib, SC558 and valdecoxib [32]. Tyr371, via its tyrosyl radical, has been reported to play a key role in the initial catalytic step of converting arachidonic acid into prostaglandin G_2_. Therefore, it can be hypothesized that the occupancy of the ligand at this specific residue may be a contributing factor to the observed anti‐inflammatory activity [33]. Furthermore, interactions with Gln178, Leu338 and Phe504 are localized within the COX‐2 side pocket, a hallmark of selective inhibitors. All selected ligands also form an extended hydrophobic network involving aromatic rings. This network spans from the catalytic lobe to the allosteric region, which is a domain unique to COX‐2‐specific drugs [34].

With regard to the 5‐LOX enzyme, it is important to consider that effective COX‐2 inhibition has the potential to redirect arachidonic acid toward alternative pathways, such as LOX‐mediated biosynthesis, potentially increasing LTs production key inflammatory and allergic mediators. This underscores the significance of the development of dual COX/LOX inhibitors.

To validate this hypothesis, molecular docking was extended to assess the selected ligands against the human 5‐LOX enzyme. The structurally complete wild‐type protein was used, with zileuton the only FDA‐approved 5‐LOX inhibitor as a reference. Docking of zileuton with 5–LOX (PDB: 3O8Y) [35] yielded a binding energy of –7.83 kcal/mol, in agreement with previously reported studies, including interactions forming hydrogen bonds with His367 and Gln363 and seven hydrophobic contacts [36].

Ligands **8**–**13** (Table 1, column 3O8Y) demonstrated favorable binding affinities. Ligand **8**, which contains a phenylalanine residue, formed two key hydrogen bonds: one with His367, associated with the ferric center and another with Ala424 both observed in the zileuton complex. Ligand **9**, which bears a tyrosine residue, was positioned near Fe^2+^ through its phenolic –OH, forming a hydrogen bond with the *C*‐terminal carboxylate of Ile673, a residue that directly coordinates the catalytic Fe^2+^. This interaction has the potential to disrupt metal coordination and hinder arachidonic acid access. The structure was stabilized by additional hydrogen bonds with Gln363, Thr364 and Ala424, along with a π‐donor interaction (2.68 Å) between the anisole ring and Asn425, complemented by alkyl and π‐alkyl hydrophobic contacts.

Ligand **10** exhibited lower affinity (binding energy <–7 kcal/mol), likely due to its position being distal from Fe^2+^, resulting in no direct interactions with residues near the metal. However, it engaged Tyr181, a key component of the FY cork, which regulates arachidonic acid access to the catalytic site [37].

Ligand **12** displayed binding energies close to –8 kcal/mol, although its electron‐donating groups did not directly interact with Fe^2+^. Nevertheless, it formed hydrogen bonds with His367 and His372, two members of the histidine triad coordinating the Fe^2+^ center, suggesting potential 5‐LOX inhibition [38].

Finally, it was observed that ligands **11** and **13** were noteworthy forming hydrogen bonds with residues such as Tyr181, Gln363, His367 (ligand **11**) and Asn425, Ala424, His367 (ligand **13**), alongside extensive nonpolar interactions. Both adopted an open U‐shaped conformation, reminiscent of arachidonic acid in crystallographic complexes. Interestingly, the aromatic ring was oriented toward Leu368, positioning carbonyl groups in proximity to the Fe^2+^ center. This configuration is analogous to that of the natural inhibitor NDGA, which has been observed to bind to Leu368 and place hydroxyl groups over Fe^2+^, in agreement with previous works [39, 40].

Subsequently to the molecular docking results, compounds **9**, **10** and **12** were selected for further investigation alongside the reference COX‐2 inhibitor celecoxib. In the case of 5‐LOX enzyme, compounds **8**, **9** and **11** were selected in addition to zileuton, a clinically approved inhibitor. The selection process was guided by two key factors: firstly, the binding affinity and secondly, the presence of key interactions that are indicative of enzymatic inhibition.

**FIGURE 2:**
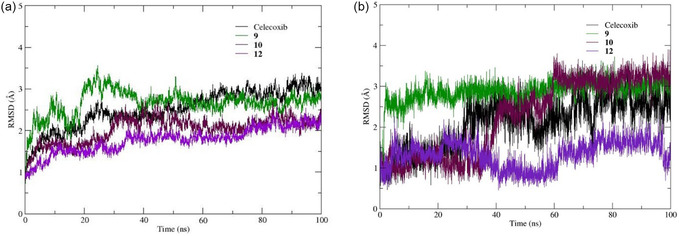
RMSD as a function of simulation time during the production stages for (a) the Cα backbone of the COX‐2 protein (PDB: 3LN1) and (b) ligands **9**, **10** and **12** aligned to the 3LN1 binding site.

For COX‐2, all ligand‐bound complexes analyzed through molecular dynamics (MD) simulations demonstrated high structural stability, as assessed by the RMSD. As shown in Figure 2a, the protein backbone trajectories of ligands **9**, **10** and **12** followed stability trends comparable to celecoxib (Figure 2a, black line), reaching equilibrium after approximately 25 ns and maintaining structural integrity with minimal fluctuations thereafter.

The relative stability of the complexes followed the order: **12** > **10** > celecoxib > **9**. Celecoxib displayed an average RMSD of 2.50 Å (range: 0.719–3.384 Å), while compound **12** showed improved stability with an average RMSD of 1.774 Å (range: 0.67–2.59 Å).

When analyzing ligand stability within the COX‐2 catalytic pocket, compounds **9** and **12** yielded the most favorable results. Ligand **9** had an average RMSD of 2.66 Å (Figure 2b, green line) and became stable after the initial 5 ns. A subtle shift in the conformation of the anisole ring was observed, adopting a folded geometry with perpendicular aromatic systems, which remained stable throughout the simulation. Ligand **12** exhibited superior conformational stability, with an average RMSD of 1.32 Å, considerably lower than that of celecoxib. Despite slight fluctuations were observed around 30 and 60 ns, the ligand maintained a stable pose, which is noteworthy given its high conformational flexibility.

This stability can be attributed to persistent hydrogen bonding interactions (Table 2, section 3LN1). Ligand **9** formed four hydrogen bonds, including a strong interaction with Arg106 (occupancy of 202%), as well as interactions with Tyr341 (30.2%), His372 (50.5%) and Ser516 (6.9%) respectively. These residues are known to be relevant in COX‐2 inhibition. In addition, ligand **12** formed six hydrogen bonds, nobably with Ser516, further supporting its potential as a selective COX‐2 inhibitor [41].

**TABLE 2:** Hydrogen bond occupancy (%) observed over 100 ns of molecular dynamics simulation for COX‐2 (PDB ID: 3LN1) with ligands **9**, **10** and **12**, and for 5‐LOX (PDB ID: 3O8Y) with ligands **8**, **9** and **11**.

Ligand	Protein					
	3LN1 (COX‐)			3O8Y (5‐LOX)		
	Donor	Acceptor	Occupacy (%)	Donor	Acceptor	Occupacy (%)
Cel	—	Leu338	54.90	—	—	—
	—	Ser339	34.88	—	—	—
	Phe504	—	44.54	—	—	—
	Ile503	—	10.44	—	—	—
Zileuton	—	—	—	Tyr181	—	79.0
	—	—	—	—	Asn425	82.50
	—	—	—	His367	—	12.80
	—	—	—	—	Leu420	7.72
**8**	‐—	—	—	His600	—	87.44
	—	—	—	Gln363	—	29.86
	—	—	—	Asn425	—	57.12
	—	—	—	Tyr181	—	66.46
**9**	Arg106	—	202.26	Thr364	—	19.15
	Tyr341	—	30.20	Gln363	—	12.65
	His372	—	60.50	His367	—	77.41
	Ser516	—	6.96	—	Ile673	36.36
	—	—	—	Asn425	—	24.95
	—	—	—	Leu368	—	20.29
	—	—	—	Thr364	—	91.19
**10**	—	Val102	5.32	—	—	—
	Tyr371	—	25.72	—	—	—
	Ser516	—	11.92	—	—	—
	—	Val74	45.60	—	—	—
**11**	—	—	—	His600	—	90.90
	—	—	—	—	Gln363	35.39
	—	—	—	—	Ile673	32.12
	—	—	—	Gln363	—	78.40
	—	—	—	Asn425	—	11.32
	—	—	—	—	Tyr181	8.94
	—	—	—	Tyr181	—	5.04
**12**	Ser516	–	39.08	—	—	—
	—	Tyr334	66.42	—	—	—
	Tyr76	—	18.58	—	—	—
	—	Val74	34.98	—	—	—
	Thr73	—	10.28	—	—	—
	Tyr108	—	20.12	—	—	—

**FIGURE 3:**
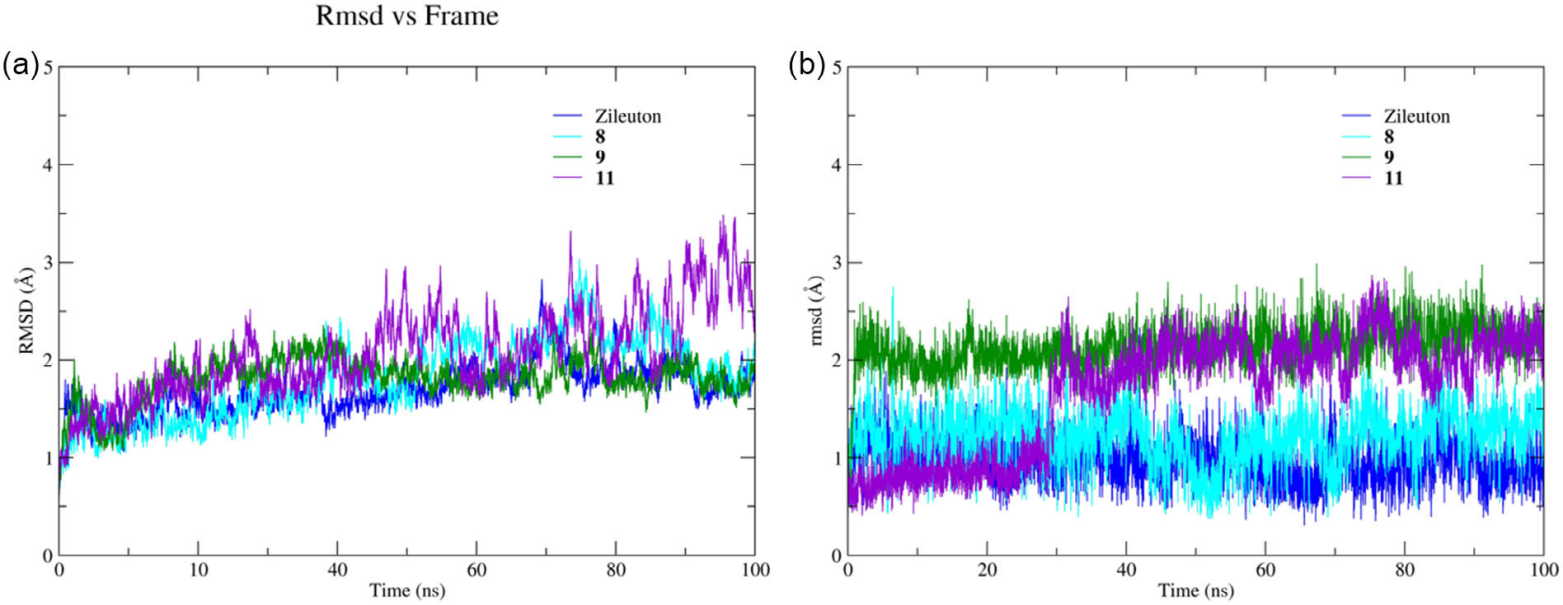
RMSD as a function of simulation time for (a) the Cα backbone of the 5‐LOX protein (PDB: 3O8Y) and (b) ligands **8**, **9** and **11** aligned to the 3O8Y binding site.

For the 5‐LOX protein, the average RMSD values for the protein backbone complexes with zileuton and ligands **8**, **9** and **11** were 1.69, 1.74, 1.81 and 2.07 Å respectively, indicating high structural stability comparable to that of reference drug (Figure 3a). This trend is more evident comparing the RMSD plots for ligands aligned to the protein, ligand **8** displayed an almost identical profile to zileuton, with an average RMSD of 1.21 Å and minimal fluctuation throughout the entire simulation trajectory (Figure 3a, blue line), ranging from 0.34 to 2.75 Å.

Ligand **9** also exhibited favorable conformational behavior (Figure 3b, green line), achieving an average RMSD of around 2.59 Å after the initial 5 ns and maintaining this consistently throughout the remainder of the simulation. Similarly, ligand **11** demonstrated comparable stability, although it stabilized slightly later, at around 25 ns and remained conformationally steady until the end of the 100 ns of simulation.

These results support the potential inhibitory activity of these ligands against 5‐LOX. Notably, ligands **9** and **10** formed hydrogen bonds with key residues, such as Tyr181, which is involved in blocking the entrance of arachidonic acid [35] and His367, which has been demonstrated by mutagenesis studies to play a critical role in 5‐LOX enzymatic studies [42, 43]. Furthermore, hydrogen bonding with the C‐terminal residue Ile673, which coordinates the catalytic Fe^2+^ ion, essential for LTs biosynthesis [44], was observed. Disruption of this coordination through ligand interaction could potentially result in enzyme inactivation (Table 2, section 3O8Y) [42].

The strong binding affinity and stability observed in molecular dynamics simulations were further supported by binding free energy calculations (Table 3). The cocrystallized reference compound celecoxib in complex with COX‐2 (PDB ID: 3LN1) exhibited a binding free energy (Δ*G*_bind_) of –34.41 kcal/mol (Table 3, entry 1). Using this as a reference, ligands **9** and **12** demonstrated improved binding affinities. Ligand **9** showed a Δ*G*_bind_ of –44.19 kcal/mol (Table 3, entry 2), primarily driven by favorable van der Waals and electrostatic interactions. This is consistent with the high hydrogen bond occupancy observed throughout the simulation.

**TABLE 3:** Predicted binding free energy and its energy components (kcal/mol) of complexes formed for ligands **9**, **10** and **12** bound to COX‐2 (3LN1).

Entry	Ligand	Δ*E*_ele_	Δ*E*_vdw_	Δ*E*_PB_	Δ_SURF_	Δ*G*_bind_
1	Celecoxib	−25.08	−47.87	7.63	30.93	−34.41
2	**9**	−24.04	−43.41	88.40	34.87	−44.19
3	**10**	−18.22	−54.63	14.16	−22.65	−17.65
4	**12**	−35.41	−63.51	15.62	44.54	−38.77

Ligand **12** (Table 3, entry 4) also exhibited enhanced binding (Δ*G*_bind_= –38.77 kcal/mol), mainly attributed to hydrophobic contacts, which are essential for enzymatic inhibition within the predominantly hydrophobic catalytic pocket of COX‐2. Additionally, a moderate contribution from electrostatic interactions (Δ*E*_ele_) further stabilized the complex.

For the 5‐LOX enzyme (3O8Y), the reference inhibitor zileuton yielded a Δ*G*_bind_ of –29.29 kcal/mol (Table 4, entry 1). In comparison, ligands **8**, **9** and **11** showed increasing stability in the order: **9** > **11** > **8** > zileuton. Ligand **9** (Table 4, entry 3) was particularly noteworthy, with a Δ*G*_bind_ of –64.27 kcal/mol, positioning it as a promising hit compound. This remarkable binding strength was predominantly attributed to van der Waals interactions and favorable polar solvation energy, which was calculated implicitly using the PBSA model.

**TABLE 4:** Predicted binding free energy and its energy components (kcal/mol) of complexes formed for ligands **8**, **9** and **11** bound to 5‐LOX (3O8Y).

Entry	Ligand	Δ*E*_ele_	Δ*E*_vdw_	Δ*E*_PB_	Δ_SURF_	Δ*G*_bind_
1	Zileuton	−28.31	−32.03	8.84	30.93	−29.29
2	**8**	84.20	−48.09	−98.78	31.73	−30.95
3	**9**	36.49	−47.98	−87.79	34.99	−64.27
4	**11**	−72.08	−72.08	14.03	47.69	−43.04

Ligand **11** (Table 4, entry 4) also demonstrated a significant Δ*G*_bind_ of –43.04 kcal/mol, primarily resulting from a balance between van der Waals and electrostatic interactions, each contributing –72.08 kcal/mol, highlighting its stable binding profile.

To elucidate the energetic contributions of ligand‐receptor interactions, a per‐residue energy decomposition analysis was conducted within an 8 Å radius of the ligand, including prosthetic groups such as heme and Fe^+2^ found in metalloproteins. In the COX‐2 enzyme (3LN1) complexed with ligand **9** (Table S1), Arg106 contributed the most to complex stabilization (–37.14 kcal/mol), primarily through electrostatic interactions, including hydrogen bonds, consistent with the high hydrogen bond occupancy observed during molecular dynamics simulations. Arg499 also displayed a notable electrostatic contribution (–9.40 kcal/mol), while residues Ser339, Tyr371, Phe504, Val509 and Ala513 stabilized the complex mainly through van der Waals forces, consistent with the hydrophobic character of the catalytic pocket. These interactions align with previously reported structural determinants of COX‐2 inhibition [32].

Ligand **11** (Table 3, entry 4), beyond exhibiting a favorable Δ*G*_bind_ (–38.77 kcal/mol), showed a balanced and consistent residue‐level energy profile throughout the simulation. This stability arose from both hydrophobic and hydrophilic contributions (Table S2) involving residues such as Val74 (–3.96 kcal/mol), His75 (–3.57 kcal/mol), Tyr76 (–3.67 kcal/mol), Tyr334 (–2.90 kcal/mol), Tyr371 (–2.28 kcal/mol) and Ser516 (–4.05 kcal/mol). Owing to its larger size, ligand **11** also engaged deeper regions of the catalytic site, forming additional hydrophobic contacts with the heme group (–2.00 kcal/mol).

In the 5‐LOX system, ligand **9** exhibited notable electrostatic interactions with His367 (–13.07 kcal/mol) and His372 (–2.08 kcal/mol), two of the three histidines involved in the coordination of the Fe^2+^ center. These interactions contributed a total of –22.36 kcal/mol, as detailed in Table S4. A similar behavior was seen for ligand **8** (Table S3), with an Fe^2+^–ligand interaction of –12.48 kcal/mol and a notable affinity from Gln363 (–10.32 kcal/mol), a residue previously implicated in selective 5‐LOX inhibition by compounds such as nordihydroguaiaretic (NDGA) [39].

For ligand **11** (Table S5), the total binding energy was driven by contributions from residues involved in arachidonic acid positioning, including Tyr181 (–3.47 kcal/mol), Gln363 (–6.20 kcal/mol), Asn425 (–2.41 kcal/mol) and His600 (–4.06 kcal/mol), with a balance of hydrophobic and electrostatic interactions [45].

Collectively, ligands **9** and **11** emerge as the most promising candidates for dual COX/LOX inhibition. To assess their drug‐likeness and potential for oral administration, compounds **8–13** were evaluated using the Protox II platform [46]. Based on Lipinski's rule of five, all ligands satisfied at least four criteria, with molecular weight being the only common violation (MW > 500 g/mol). Their predicted lipophilicity aligned well with that of commercially available NSAIDs. Furthermore, toxicity predictions indicated no hepatotoxic, carcinogenic, immunotoxic, mutagenic or cytotoxic liabilities. All ligands exhibited favorable and safe theoretical LD_50_ values, supporting their potential for further development (Table 5).

**TABLE 5:** Predicted drug‐likeness parameters and toxicity profiles of compounds 8–13 based on Lipinski's rules and Protox II evaluation.

Ligand	Predicted LD_50_ mg/kg	Predicted toxicity Clss	Hepatotoxicity	Carcinogenicity	Immunotoxicity	Mutagenicity	Cytotoxicity
**8**	1000	4	Inactive (0.74)	Inactive (0.69)	Inactive (0.99)	Inactive (0.74)	Inactive (0.70)
**9**	1000	4	Inactive (0.74)	Inactive (0.69)	Inactive (0.99)	Inactive (0.74)	Inactive (0.70)
**10**	5300	6	Inactive (0.72)	Inactive (0.71)	Inactive (0.99)	Inactive (0.76)	Inactive (0.68)
**11**	5300	6	Inactive (0.71)	Inactive (0.69)	Inactive (0.99)	Inactive (0.74)	Inactive (0.87)
**12**	5300	6	Inactive (0.72)	Inactive (0.71)	Inactive (0.99)	Inactive (0.76)	Inactive (0.68)
**13**	5300	6	Inactive (0.72)	Inactive (0.71)	Inactive (0.99)	Inactive (0.76)	Inactive (0.68)

## 3 Conclusion

The successful synthesis of hybrid compounds **8–13** suggests that they could be evaluated in vitro and further developed as anti‐inflammatory agents targeting COX‐2 and 5‐LOX. Theoretical studies revealed binding affinities and interaction profiles that are comparable to (or exceeding) that of the reference drug, positioning these ligands as promising candidates for dual inhibition of two key enzymes implicated in chronic degenerative diseases such as rheumatoid arthritis, asthma and cancer. Among them, compounds **9** (methyl (4‐(4‐methoxyphenyl)‐4‐oxobutanoyl)‐*L*‐tyrosylate) and **12** (methyl (4‐(4‐methoxyphenyl)‐4‐oxobutanoyl)‐*L*‐tyrosyl‐*L*‐phenylalaninate) emerged as lead molecules, exhibiting favorable interactions with both targets. These interactions were largely driven by the hydrophobic methoxyphenyl‐oxobutanoyl moiety and the hydrogen‐bonding capability of the tyrosine –OH group. In certain cases, electrostatic interactions with the prosthetic groups of oxidoreductase enzymes further stabilized the complexes. Overall, these findings provide a compelling rationale for further in vitro and in vivo investigation of these dual‐acting candidates.

## 4 Experimental Section

### 4.1 Materials and Methods

Column chromatography was performed using Merck silica gel (70–230 mesh) and analytical thin‐layer chromatography (TLC) was conducted on Merck 60‐F254 plates, with visualization under UV light and iodine vapor. Melting points were determined on a Fischer model 1237 apparatus and are uncorrected. Infrared (IR) spectra were recorded on a Thermo Scientific Nicolet iS10 spectrometer. ^1^H and ^13^C NMR spectra were acquired on a Varian Mercury Plus spectrometer operating at 400 and 100MHz. Mass spectra (MS) were obtained via electron impact (EI) using a Thermo Scientific ISQ CT instrument. High‐resolution mass spectrometry (HRMS) data were collected using a Bruker Maxis Impact ESI‐QTOF‐MS. Hydroxyketone intermediates were synthesized using a CEM Discover microwave reactor (Model 201A15, 20 MHz).

### 4.2 Synthesis

#### 4.2.1 Heck Reaction Procedure

A mixture of aryl halide (1.0 equiv), *N*,*N*‐diisopropylethylamine (3.0 equiv) and 2,3‐dihydrofuran (5.1 equiv) in DMF (3 mL) with the addition of H_2_O (0.2 mL) was treated with *trans*‐PdCl_2_(Gly)_2_ (5 mol%) as catalyst. The reaction mixture was placed in a microwave reactor equipped with a condenser and irradiated at 60 W for 30 min.

##### 4.2.1.1 Synthesis of 4‐Hydroxy‐1‐(4‐Methoxyphenyl)‐Butan‐1‐one (1) and 4‐Hydroxy‐1,4‐Bis(4‐Methoxyphenyl)butan‐1‐one (2)

After completion, the crude mixture was purified by column chromatography using a *n*‐hexane/ethyl acetate (7:3, v/v) eluent system, yielding two products. Compound **1** was obtained as a white solid (84 mg, 60% yield; mp 104–106°C). Compound **2** was isolated as a crystalline solid (42 mg, 31% yield; mp 67–69°C). Compound **1**: ^1^H NMR (400 MHz; CDCl_3_; TMS) δ ppm: 7.97 (d, *J* = 9.0 Hz, 2H, Ar‐H), 6.94 (d, *J* = 8.9 Hz, 2H, Ar‐H), 3.87 (s, 3H, OCH_3_), 3.74 (t, *J* = 6.0 Hz, 2H, CH_2_), 3.09 (t, *J* = 6.9 Hz, 2H, CH_2_), 2.15 (s, 1H, OH), 2.01 (m, 2H, CH_2_); ^13^C NMR (100 MHz; CDCl_3_; TMS) δ ppm: 199.2, 163.5, 130.4, 129.8, 113.7, 62.4, 55.4, 35.0, 27.0. m/z: 194 (M^+^, 4%), 176(83), 150(79), 135(100), 107(26), 92(22); IR (ATR, cm^−1^) ν: 3357, 2932, 1676, 1596, 1361, 1018, 827, 764. Compound **2**: ^1^H NMR (400 MHz; CDCl_3_; TMS) δ ppm: 7.93 (d, *J* = 9.0 Hz, 2H, Ar‐H), 7.30 (d, *J* = 8.8 Hz, 2H, Ar‐H), 6.92 (d, *J* = 9.0 Hz, 2H, Ar‐H), 6.89 (d, *J* = 8.7 Hz, 2H, Ar‐H), 4.77 (t, *J* = 6.3 Hz, 1H, CH), 3.87 (s, 3H, OCH_3_), 3.81 (s, 3H, OCH_3_), 3.05 (dd, *J* = 7.6, 6.9 Hz, 2H, CH_2_), 2.46 (s, 1H, OH), 2.17 (m, 2H CH_2_); ^13^C NMR (100 MHz; CDCl_3_; TMS) δ ppm: 199.2, 163.5, 159.0, 136.5, 130.4, 129.9, 127.0, 113.8, 113.8, 73.3, 55.4, 55.3, 34.5, 33.2; MS (EI, 70 eV) m/z: 299 (M‐18, 2%), 280(79), 265(87), 164(44), 151(50), 135(100), 92(12); IR (ATR, cm^−1^) ν: 3315, 2953, 2848, 1663, 1 597 1242, 1169, 1021, 833, 808.

Synthetic compound **1a** was successfully identified and characterized using mass spectrometry; m/z: 176 (M+, 67%), 160(14), 136(100), 103(16), 77(33) [47].

##### 4.2.1.2 Synthesis of 4‐(4‐Methoxyphenyl)‐4‐Oxobutanoic Acid (3)

Compound **1** (0.080 g, 0.41 mmol) was dissolved in dry DMF and stirred over activated 3 Å molecular sieves. Pyridinium dichromate (PDC; 0.46 g, 1.2 mmol) was added and the reaction mixture was stirred at ambient temperature for 24 h. Upon completion, the reaction was quenched with an aqueous solution of sodium thiosulfate (Na_2_S_2_O_3_), followed by extraction with ethyl acetate (3 x 20 mL). The combined organic layers were washed with water, dried over anhydrous Na_2_SO_4_ and concentrated under reduced pressure. The crude product was purified by flash chromatography using a *n*‐hexane/ethyl acetate (7:3, v/v) solvent system, affording compound **3** as a white solid (0.070 g, 82% yield; mp. 140–142°C). The spectroscopic and analytical data were consistent with those previously reported in the literature [29]. ^1^H NMR (400 MHz; CDCl_3_; TMS) δ ppm: 7.97 (d, *J* = 8.9 Hz, 2H, Ar‐H), 6.94 (d, *J* = 8.9 Hz, 2H, Ar‐H), 3.88 (s, 3H, OCH_3_), 3.28 (t, *J* = 6.6 Hz, 2H, CH_2_), 2.80 (t, *J* = 6.6 Hz, 2H, CH_2_); ^13^C NMR (100 MHz; CDCl_3_; TMS) δ ppm: 196.3, 177.9, 163.6, 130.3, 129.4, 113.7, 55.4, 32.7, 28.0; MS (EI, 70 eV) m/z: 208 (M^+^, 24%), 136(20), 135(100), 107(18), 92(16), 77(32); IR (ATR, cm^−1^) ν: 3366, 2922, 1582, 1483, 1426, 1210, 820.

#### 4.2.2 Preparation of Methyl Esters

To a suspension of the corresponding amino acid (1.0 equiv) in methanol, trimethylsilyl chloride (TMSCl; 3.0 equiv) was added. The reaction mixture was heated under reflux for 4 h and then stirred at room temperature overnight. After completion, the solvent was removed under reduced pressure using a rotary evaporator. The resulting residue was recrystallized from methanol to afford the corresponding amino acid methyl ester hydrochloride salt.

##### 4.2.2.1 L‐Phenylalanine Methyl Ester Hydrochloride (4)

Recrystallization of the crude reaction mixture obtained from 1.3 mmol of *L*‐phenylalanine afforded the title compound as white needle‐like crystals (0.293 g; 90% yield; mp 111–112°C); ^1^H NMR (400 MHz, DMSO‐d_6_) δ ppm: 7.32–7.19 (m, 5H, Ar‐H), 4.18 (dd, *J* = 7.5, 5.6 Hz, 1H, CH), 3.60 (s, 3H, OCH_3_), 3.21 (dd, *J* = 13.9, 5.5 Hz, 1H), 3.07 (dd, *J* = 13.8, 7.7 Hz, 1H); ^13^C NMR (100 MHz, DMSO‐d_6_, TMS) δ 170.0, 135.4, 130.0, 129.2, 127.9, 53.9, 53.1, 36.5; IR (ATR, cm^−1^) ν: 3213, 3026, 2853, 1732, 1480, 857, 743.

##### 4.2.2.2 L‐Tyrosine Methyl Ester Hydrochloride (5)

Recrystallization of the crude reaction mixture obtained from 1.1 mmol of *L*‐tyrosine afforded the title compound as a white solid (0.268 g, 84% yield; mp 192C–194°C); ^1^H NMR (400 MHz, DMSO‐d_6_, TMS) δ ppm: 9.52 (s, 1H, OH), 8.61 (s, 2H, NH), 6.98 (d, *J* = 8.4 Hz, 2H, Ar‐H), 6.70 (d, *J* = 8.4 Hz, 2H), 4.11 (dd, *J* = 6.4, 6.4 Hz, 1H), 3.63 (s, 3H, OCH_3_), 3.09–2.92 (m, 2H, CH_2_); ^13^C NMR (100 MHz, DMSO‐d_6_) δ ppm: 170.0, 157.3, 131.1, 131.0, 125.0, 116.1, 116.0, 54.1, 53.2, 35.7; MS (EI, 70 eV) m/z: 195(M^+^, 9), 136, 107(100), 91(12), 88(35), 77(11); IR (ATR, cm^−1^) ν: 3350, 3300, 2933, 1742, 1596, 1257, 1018, 837.

#### 4.2.3 General Procedure for Coupling

To a solution of the corresponding methyl ester (1.1 equiv) in a THF/DMSO (9:1, v/v) mixture, 2.5 equivalents of *N*,*N*‐diisopropylethylamine (DIPEA) were added and the reaction mixture was stirred at 0°C for 1 h. In parallel, a solution of the δ‐keto acid or δ‐keto acid‐amino acid conjugate (1 equiv) in THF was preactivated with *i*‐butylchloroformate (*i*‐BuOCOCl; 1.1 equiv) and DIPEA (2 equiv) under stirring at 0°C. The ester solution was then added dropwise to the activated acid mixture. The reaction was stirred for 1 h at 0°C, followed by 18 h at room temperature. Upon completion, the reaction mixture was concentrated under reduced pressure and the residue was extracted with ethyl acetate (3 × 50 mL). The combined organic layers were washed successively with 10% aqueous HCl and saturated NaHCO_3_, dried over anhydrous Na_2_SO_4_, filtered and concentrated. The crude product was purified by flash chromatography as specified for each compound.

##### 4.2.3.1 Methyl (4‐(4‐Methoxyphenyl)‐4‐Oxobutanoyl)phenylalaninate (6)

The coupling reaction was performed using 0.070 g of δ‐keto acid **3** (0.33 mmol) and 0.079 g of methyl ester **4** (0.37 mmol) following the general procedure. Purification by column chromatography (*n*‐hexane/ethyl acetate, 9:1, v/v) afforded compound **6** as a white solid (0.097 g, 77% yield; mp 140–142°C); ^1^H NMR (400 MHz, CDCl_3_, TMS) δ ppm: 7.93 (d, *J* = 8.8 Hz, 2H, Ar‐H), 7.31–7.20 (m, 2H, Ar‐H), 7.11 (d, *J* = 6.4 Hz, 2H, Ar‐H), 6.92 (d, *J* = 8.8 Hz, 2H, Ar‐H), 6.23 (d, *J* = 7.4 Hz, 1H, N‐H), 4.94–4.84 (m, 1H, CH), 3.87 (s, 3H, CH_3_), 3.71 (s, 3H, CH_3_), 3.33–3.20 (m, 2H, CH_2_), 3.18–3.04 (m, 2H, CH_2_), 2.71–2.52 (m, 2H, CH_2_); ^13^C NMR (100 MHz, CDCl_3_, TMS) δ ppm: 197.3, 172.1, 172.0, 163.8, 136.0, 130.5, 129.8, 129.5, 128.7, 127.2, 113.9, 55.7, 53.4, 52.5, 38.1, 33.7, 30.3; MS (EI, 70 eV) m/z: 369 (M^+^, 4%), 207(38), 191(100), 163(9), 135(87). IR (ATR, cm^−1^) ν: 3339, 2942, 2842, 1737, 1650, 1524, 1180, 985, 830, 697; HRMS (ESI^+^) m/z: calcd for [M + H]^+^ C_30_H_32_N_2_O_8_ 370.1654; found 370.1700.

##### 4.2.3.2 Methyl (4‐(4‐Methoxyphenyl)‐4‐Oxobutanoyl)tyrosinate (7)

The coupling reaction was performed using 0.070 g of δ‐keto acid **3** (0.33 mmol) and 0.085 g of methyl ester **5** (0.37 mmol) following the general procedure. Purification by column chromatography (*n*‐hexane/ethyl acetate, 8:2, v/v) afforded compound **7** as an amorphous white solid (89 mg, 69% yield; mp 165–167°C); ^1^H NMR (400 MHz, CDCl_3_, TMS) δ ppm: 7.93 (d, *J* = 8.9 Hz, 2H, Ar‐H), 6.94 (dd, *J* = 11.2, 8.7 Hz, 4H, Ar‐H), 6.69 (d, *J* = 8.5 Hz, 2H, Ar‐H), 6.62 (s, 1H, OH), 6.41 (d, *J* = 8.0 Hz, 1H, NH), 4.85 (dd, *J* = 13.9, 6.1 Hz, 1H, CH), 3.86 (s, 3H, CH_3_), 3.72 (s, 3H, CH_3_), 3.34–3.16 (m, 2H, CH_2_), 3.02 (ddd, *J* = 20.4, 14.0, 6.0 Hz, 2H, CH_2_), 2.73–2.54 (m, 2H, CH_2_); MS (EI, 70 eV) m/z: 385 (M^+^, 2), 208(24), 191(100), 178(12), 163 (5), 135(34); IR (ATR, cm^−1^) ν: 3336, 2923, 2854, 1739, 1651, 1513, 1243, 1026, 831; HRMS (ESI^+^) m/z: calcd for [M + H]^+^ C_30_H_32_N_2_O_8_ 386.1604; found 386.1640.

##### 4.2.3.3 Methyl 4‐(4‐Methoxyphenyl)‐4‐Oxobutanoyl‐L‐Tyrosyl‐L‐Tyrosinate (10)

The coupling reaction was performed using 0.050 g of δ‐keto acid‐amino acid **9** (0.13 mmol) and 0.034 g of methyl ester **5** (0.15 mmol) following the general procedure. Purification by column chromatography (*n*‐hexane/ethyl acetate, 6:4, v/v) afforded compound **10** as an a white solid (0.059 g, 80% yield), mp 170–171°C; ^1^H NMR (400 MHz, CD_3_OD, TMS) δ ppm: 7.94 (dd, *J* = 8.8, 4.4 Hz, 2H, Ar‐H), 7.14–6.86 (m, 6H, Ar‐H), 6.67 (t, J = 7.0 Hz, 4H, Ar‐H), 4.56 (ddd, *J* = 14.9, 11.5, 5.8 Hz, 2H, CH), 3.67 (s, 1H, CH_3_), 3.85 (d, *J* = 2.7 Hz, 3H, CH_3_), 3.58 (s, 2H, CH_3_), CH_3_, 3.25–3.15 (m, 2H, CH_2_), 3.09–2.83 (m, 2H, CH_2_), 2.69 (dt, *J* = 22.0, 11.2 Hz, 1H, CH_2_), 2.59–2.40 (m, 1H, CH_2_). ^13^C NMR (100 MHz, CD_3_OD, TMS) δ ppm: 199.5, 175.1, 173.6, 173.1, 165.3, 157.3, 157.2, 131.5, 131.5, 131.3, 131.2, 130.7,129.1, 129.1, 128.6, 128.5, 116.2, 116.1, 114.8, 56.1, 56.0, 55.5, 52.6, 37.6, 34.7, 34.6, 30.8; MS (DIP–EI, 70 eV) m/z: 548 (M^+^, 1%), 354(5), 208(21), 191(100), 164(13), 135(44), 107(37); IR (ATR, cm^−1^) ν: 3430, 3303, 2937, 1736, 1676, 1665, 1642, 1536, 1361, 1266, 1221, 1176, 832, 748, 697; HRMS (ESI^+^) m/z: *calcd for* [M+H]^+^ C_30_H_32_N_2_O_8_ 549.2237; found 549.2266.

##### 4.2.3.4 Methyl (4‐(4‐Methoxyphenyl)‐4‐Oxobutanoyl)‐L‐Phenylalanyl‐L‐Tyrosinate (11)

The coupling reaction was performed using 0.050 g of δ‐keto acid‐amino acid **8** (0.14 mmol) and 0.035 g of methyl ester **5** (0.15 mmol) following the general procedure. Purification by column chromatography (*n*‐hexane/ethyl acetate, 1:1, v/v) afforded compound **11** as an a white solid (0.054 g, 73% yield); mp 130–131°C; ^1^H NMR (400 MHz, CD_3_OD, TMS) δ ppm: 7.96–7.90 (m, 2H, Ar‐H), 7.26–7.10 (m, 5H, Ar‐H), 7.03–6.90 (m, 4H, Ar‐H), 6.71–6.64 (m, 2H, Ar‐H), 4.61 (dq, *J* = 9.7, 6.1 Hz, 2H, CH), 3.84 (d, *J* = 2.9 Hz, 3H, CH_3_), 3.66 (s, 1H, CH_3_), 3.57 (s, 2H, CH_3_), 3.27–2.71 (m, 6H, CH_2_), 2.49 (dtd, *J* = 21.5, 15.1, 6.4 Hz, 2H, CH_2_); ^13^C NMR (100 MHz, CD_3_OD) δ ppm: 199.5, 175.0, 173.5, 173.1, 165.3, 157.4, 157.3, 138.5, 131.5, 131.3, 130.7, 130.2, 129.3, 127.6, 116.2, 114.8, 56.0, 55.8, 55.5, 52.6, 38.4, 37.6, 34.6, 30.7; MS (DIP‐EI, 70 eV) m/z: 532 (M^+^, 1%), 355(18), 338(25), 191(100), 163(5), 135(26), 107(11); IR (ATR, cm^−1^) ν: 3303, 2920, 2726, 1660, 1643, 1600, 1513, 1256, 1175, 1056, 829; HRMS (ESI^+^) m/z: calcd for [M + H]^+^ C_30_H_33_N_2_O_7_ 533.2288; found 533.2304.

##### 4.2.3.5 Methyl (4‐(4‐Methoxyphenyl)‐4‐Oxobutanoyl)‐L‐Tyrosyl‐L‐Phenylalaninate (12)

The coupling reaction was performed using 0.050 g of δ‐keto acid‐amino acid **9** (0.13 mmol) and 0.031 g of methyl ester **4** (0.14 mmol) following the general procedure. Purification by column chromatography (*n*‐hexane/ethyl acetate 1:1) afforded compound **12** as an a white solid (0.055 g, 77% yield); mp 144–145°C); ^1^H NMR (400 MHz, CD_3_OD, TMS) δ ppm: 8.19 (d, *J* = 7.9 Hz, 1H, NH), 8.15 (d, *J* = 8.1 MHz, 1H, NH), 7.95 (d, *J* = 8.9 Hz, 2H, Ar‐H), 7.28–7.15 (m, 5H, Ar‐H), 7.01 (dd, *J* = 8.7, 6.9 Hz, 4H, Ar‐H), 6.66 (d, *J* = 8.4 Hz, 2H, Ar‐H), 4.65 (td, *J* = 8.4, 5.8 Hz, 1H, CH), 4.56–4.47 (m, 1H, CH), 3.86 (s, 3H, CH_3_), 3.63 (s, 1H, CH_3_), 3.58 (s, 2H, CH_3_), 3.24–3.11 (m, 3H, CH_2_), 3.07–2.97 (m, 2H, CH_2_), 2.69 (dd, *J* = 14.1, 9.2 Hz, 1H, CH_2_), 2.62–2.39 (m, 2H, CH_2_). ^13^C NMR (100 MHz, CD_3_OD) δ 199.6, 175.1, 173.7, 173.0, 165.4, 157.1, 138.0, 131.5, 131.2, 130.7, 130.3, 129.5, 129.1, 127.8, 116.1, 114.8, 56.1, 56.0, 55.2, 52.6, 38.3, 37.7, 34.7, 30.8; MS (DIP‐EI, 70 eV) m/z: 532(M^+^, 1%), 190(24), 162(8), 150(), 135(100), 120(24), 91(52), 77(42); IR (ATR, cm^−1^) ν: 3438, 3316, 2925, 2853, 1732, 1677, 1650, 1601, 1258, 1223, 1175, 1030, 829, 698; HRMS (ESI^+^) m/z: calcd for [M + H]^+^ C_30_H_33_N_2_O_7_ 533.2288; found 533.2301.

##### 4.2.3.6 Methyl (4‐(4‐Methoxyphenyl)‐4‐Oxobutanoyl)‐L‐Phenylalanyl‐L‐Phenylalaninate (13)

The coupling reaction was performed using 0.050 g of δ‐keto acid‐amino acid **8** (0.14 mmol) and 0.033 g of methyl ester **4** (0.15 mmol) following the general procedure. Purification by recrystallization afforded compound **13** as an a white solid (0.031 g, 44% yield); mp 198–199°C decomp. ^1^H NMR (400 MHz, DMSO) δ ppm: 8.41 (d, *J* = 7.6 Hz, 1H, N‐H), 8.12 (d, *J* = 7.7 Hz, 1H, N‐H), 7.88 (d, *J* = 8.6 Hz, 6H), 7.22 (qd, *J* = 11.6, 4.4 Hz, 35H), 7.02 (d, *J* = 8.8 Hz, 8H), 4.48 (dd, *J* = 14.9, 5.6 Hz, 1H), 3.82 (s, 12H), 3.55 (s, 15H), 3.08–2.91 (m, 15H), 2.68 (dd, *J* = 13.7, 9.7 Hz, 1H), 2.38 (dd, *J* = 13.0, 6.7 Hz, 3H). MS (DIP‐EI, 70 eV) m/z: 516(M^+^, 2%), 338(1), 279(13), 246(14), 191(45), 167(39), 149(100), 135(47), 120(23), 91(58); IR (ATR, cm^−1^) ν: 3214, 3084, 2967, 2927, 1673, 1659, 1460, 1337, 1136, 756, 699; HRMS (ESI^+^) m/z: calcd for [M + H]^+^ C_30_H_33_N_2_O_6_ 517.2338; found *517.2343.*

#### 4.2.4 General Procedure for Methyl Ester Hydrolysis

To a round‐bottom flask containing 1 equivalent of the corresponding methyl ester dissolved in a mixture of THF/H_2_O/MeOH (25:3:0.1), a 2 M aqueous solution of lithium hydroxide (LiOH, 3 equiv) was added dropwise under ice‐bath cooling. The reaction mixture was stirred at 0°C for 4 h. Upon completion, the reaction was concentrated to dryness under reduced pressure. The residue was resuspended in CH_2_Cl_2_ and acidified to pH 3 with 1 N HCl. The organic phase was dried over anhydrous Na_2_SO_4_, filtered and evaporated under reduced pressure to afford the corresponding carboxylic acid.

##### 4.2.4.1 (4‐(4‐Methoxyphenyl)‐4‐Oxobutanoyl)‐L‐phenylalanine (8)

The hydrolysis reaction was performed using 0.088 g of ester **6** (0.23 mmol) and 0.029 g of LiOH (0.71 mmol) following the general procedure. Purification by recrystallization afforded compound **8** as an a white solid, (0.069 g, 81% yield); mp 168–169°C. ^1^H NMR (400 MHz, CD_3_OD TMS) δ ppm: 7.90 (d, *J* = 8.8 Hz, 2H), 7.16 (m, 5H), 6.95 (d, *J* = 8.8 Hz, 2H), 4.53 (dd, *J* = 8.0, 4.9 Hz, 1H), 3.83 (s, 3H), 3.21 (dd, *J* = 13.8, 4.8 Hz, 1H), 3.13 (td, *J* = 6.9, 3.9 Hz, 2H), 2.95 (dd, *J* = 13.7, 8.1 Hz, 1H), 2.52 (t, *J* = 6.9 Hz, 2H). ^13^C NMR (100 MHz, CD_3_OD) δ ppm: 199.1, 174.8, 165.2, 138.4, 131.4, 130.8, 130.3, 129.3, 127.7, 114.8, 56.0, 55.1, 38.5, 34.5, 30.7; MS (EI, 70 eV) m/z: 372(M + H, 2%), 190.08(36), 161(38), 147(20), 135(100), 92(17), 77(25); IR (ATR, cm^−1^) ν: 3323, 2924, 2850, 1644, 1 597, 1574, 1416, 1250, 1168, 1028, 979, 830, 700; HRMS (ESI^+^) m/z: calcd for [M + H]^+^ C_20_H_22_NO_5_ 356.1498; found 356.1400.

##### 4.2.4.2 (4‐(4‐Methoxyphenyl)‐4‐Oxobutanoyl)‐L‐tyrosine (9)

The hydrolysis reaction was performed using 0.089 g of ester **7** (0.23 mmol) and 0.029 g of LiOH (0.69 mmol) following the general procedure. Purification by recrystallization afforded compound **9** as an a light yellow solid, (0.073 g, 85% yield); mp 184–185°C. ^1^H NMR (400 MHz, CDCl_3_, TMS) δ ppm: 7.93 (d, *J* = 8.9 Hz, 2H), 7.05 (d, *J* = 8.5 Hz, 2H), 6.98 (d, *J* = 8.9 Hz, 2H), 6.69 (d, *J* = 8.5 Hz, 2H), 4.61 (dd, *J* = 8.6, 5.3 Hz, 1H), 3.85 (s, 3H), 3.15 (t, *J* = 7.0 Hz, 2H), 3.09 (dd, *J* = 14.0, 5.2 Hz, 1H), 2.86 (dd, *J* = 14.0, 8.6 Hz, 1H), 2.56 (td, *J* = 7.0, 1.6 Hz, 1H). ^13^C NMR (100 MHz, CD_3_OD) δ ppm: 199.1, 174.9, 174.8, 165.2, 157.2, 131.4, 131.3, 130.8, 129.0, 116.1, 114.8, 56.0, 55.2, 37.7, 34.5, 30.7; MS (EI, 70 eV) m/z: 372(M + H, 2%), 190.08(36), 161(38), 147(20), 135(100), 92(17), 77(25); IR (ATR, cm^−1^) ν: 3321, 2919, 1725, 1644, 1513, 1244, 1168, 1025, 978, 828; HRMS (ESI^+^) m/z: *calcd for* [M + H]^+^ C_20_H_22_NO_6_ 372.1447; found 372.1453.

## 5 Computational Details

First, conformational searches for ligands **8–13** were performed using Spartan´20 software [48], and the most populated conformers were selected for a geometry optimization at the HF/6‐311+G(d, p) level of theory using the Gaussian16 software [49].

Molecular docking studies were carried out using the optimized structures of ligands **8–13** against the human COX‐2 enzyme, downloaded from Protein Data Bank (PDB ID: 3LN1), which contains cocrystalized celecoxib compound in the active site and 5‐LOX (PDB ID: 3O8Y). For both proteins, subunit A was retained, water molecules were removed and polar hydrogens were added using *prepare_receptor.py* from MGLTools version 1.5.6 [50]. Ligands were prepared with *prepare_ligand.py* script [50]. Docking grid parameters were defined using AutoGridFR v1.2 [51], centered on the celecoxib binding site for COX‐2. The coordinates of the center X, Y and Z were 30.29, −23.07 and −15.99, respectively, with box dimensions of X, Y, Z: 50, 50, 50 for 3LN1. For 5‐LOX, coordinates were taken from literature reports and established as X, Y, Z: −2.24, 25.69, −0.94, in a box of 60, 66 and 60 with a grid spacing of 0.375 for both proteins [36].

Docking was performed using AutoDock 4.2 [50] with default parameters: population of 5000 individuals. 100,000,000 evaluations, 500,000 generations and a maximum number of 5000 iterations. Docking poses were visualized using The Discovery Studio software [52] y USCF Chimera [53].

The stability and evolution of the drug–receptor interaction over time from the best docking poses were analyzed using molecular dynamics simulations with NAMD software [54]. Parameterization of the complex was performed using CHARMM‐GUI [55] and Solution Builder [56, 57] and the CHARMM36 force field [58]. The pH was set to 7.4 in the generated cubic cell and the solvation was applied with the TIP3 water model. Ions of sodium (Na^+^) and chloride (Cl^−^) were added to achieve charge neutrality and electrostatics were addressed through the implementation of the Particle Mesh Ewald (PME) method [59].

The minimization process was conducted using the conjugate gradient method, followed by heating from 0 to 310 K over 500 ps and equilibration for 500 ps using the NVT ensemble with Langevin dynamics [60]. The system was further equilibrated under NPT conditions (310 K, 1 atm) during 5 ns and a 100 ns production simulation was performed using a 2 fs time step for each system.

The trajectory analysis included RMSD calculations and hydrogen bond were done using the VMD software [61], while binding free energy (Δ*G*_bind_) was estimated from the last 40 ns of the trajectory using the MM/PBSA approach implemented in gmx_MMPBSA program [62].

## Supporting Information

Additional supporting information can be found online in the Supporting Information section. **Supporting** **Fig. S1**: ^1^H NMR spectrum of compound **1** (400 MHz, CDCl_3_). **Supporting** **Fig. S2**: ^13^C NMR spectrum of compound **1** (100 MHz, CDCl_3_). **Supporting** **Information** **Fig. S3**: ^1^H NMR spectrum of compound **2** (400 MHz, CDCl_3_). **Supporting** **Fig. S4**: ^13^C NMR spectrum of compound **2** (100 MHz, CDCl_3_). **Supporting** **Fig. S5**: ^1^H NMR spectrum of compound **3** (400 MHz, CDCl_3_). **Supporting** **Fig. S6**: ^13^C NMR spectrum of compound **3** (100 MHz, CDCl_3_). **Supporting Fig. S7**: ^1^H NMR spectrum of compound **4** (400 MHz, DMSO‐*d*_6_). **Supporting** **Fig. S8**: ^13^C NMR spectrum of compound **4** (100 MHz, DMSO‐*d*_6_). **Supporting** **Fig. S9**: ^1^H NMR spectrum of compound **5** (400 MHz, DMSO‐*d*_6_). **Supporting** **Fig. S10**: ^13^C NMR spectrum of compound **5** (100 MHz, DMSO‐*d*_6_). **Supporting** **Fig. S11**: ^1^H NMR spectrum of compound **6** (400 MHz, CDCl_3_). **Supporting** **Fig. S12**: ^13^C NMR spectrum of compound **6** (100 MHz, CDCl_3_). **Supporting** **Fig. S13**: ^1^H NMR spectrum of compound **7** (400 MHz, CDCl_3_). **Supporting** **Fig. S14**: ^13^C NMR spectrum of compound **7** (100 MHz, CDCl_3_). **Supporting** **Fig. S15**: ^1^H NMR spectrum of compound **8** (400 MHz, CDCl_3_). **Supporting** **Fig. S16**: ^13^C NMR spectrum of compound **8** (100 MHz, CDCl_3_). **Supporting** **Fig. S17**: ^1^H NMR spectrum of compound **9** (400 MHz, CDCl_3_). **Supporting** **Fig. S18**: ^13^C NMR spectrum of compound **9** (100 MHz, CDCl_3_)_._ **Supporting** **Fig. S19**: ^1^H NMR spectrum of compound **10** (400 MHz, CD_3_OD). **Supporting** **Fig. S20**: ^13^C NMR spectrum of compound **10** (100 MHz, CD_3_OD). **Supporting** **Fig. S21**: ^1^H NMR spectrum of compound **11** (400 MHz, CD_3_OD). **Supporting** **Fig. S22**: ^13^C NMR spectrum of compound **11** (100 MHz, CD_3_OD). **Supporting** **Fig. S23**: ^1^H NMR spectrum of compound **12** (400 MHz, CD_3_OD). **Supporting** **Fig. S24**: ^13^C NMR spectrum of compound **12** (100 MHz, CD_3_OD).**Supporting** **Fig. S25**: ^1^H NMR spectrum of compound **13** (400 MHz, DMSO‐*d*_6_). **Supporting** **Fig. S26**: Observed interactions with protein 3LN1 and the ligands: a) **8**, b) **9**, c) **10**, d) **11**, e) **12**, and f) **13**. **Supporting** **Fig. S27**: Observed interactions with protein 3O8Y and the ligands: a) **8**, b)**9**, c)**10**, d)**11**, e)**12** y f)**13**. **Supporting** **Table S1**: Per‐residue energy decomposition complex 3LN1 and compound **9**. **Supporting** **Table S2**: Per‐residue energy decomposition complex 3LN1 and compound **12**. **Supporting** **Table S3**: Per‐residue energy decomposition complex 3O8Y and compound **8**. **Supporting** **Table S4**: Per‐residue energy decomposition complex 3O8Y and compound **9**. **Supporting** **Table S5**: Per‐residue energy decomposition complex 3O8Y and compound **11**.

The authors have cited additional references within the Supporting Information [30, 31]. Please include SI references with consecutive numbering directly after the last manuscript reference: 1, 2, 3,…30, 31.

## Funding

This work was supported by the Secretaría de Ciencia, Humanidades, Tecnología e Innovación (SECIHTI, formerly CONAHCYT, México) (252239, 294024, IIXM 4933)and the Coordinación de la Investigación Científica Universidad Michoacana de San Nicolás de Hidalgo.

## Conflicts of Interest

The authors declare no conflicts of interest.

## Supporting information

Supplementary Material

## Data Availability

The data that support the findings of this study are available from the corresponding author upon reasonable request.
