# Supplementary material for: Dual Inhibition of COX‐2/5‐LOX Through Novel Hybrids of NSAIDs and Peptides: Insights from Molecular Dynamics Simulation and Per‐Residue Decomposition
Source: ChemistryOpen. 2026 Mar 20;15(3):e202500452. doi: 10.1002/open.202500452 (PMC13098134; doi:10.1002/open.202500452)
Supplement: Supplementary file 1 — Supplementary Material [file OPEN-15-e202500452-s001.pdf]

Supporting information

Dual Inhibition of COX-2/5-LOX Through Novel Hybrids of NSAIDs and Peptides: Insights from Molecular Dynamics Simulation and Per-Residue Decomposition

J. Carlos Jiménez-Cruz[1], Ramón Guzmán-Mejía[2], Pedro Navarro-Santos[1*], Hugo A. García-Gutiérrez[2], Julio C. Ontiveros-Rodríguez[1], Rafael Herrera-Bucio[2], Verónica Cortés-Muñoz[2] and Judit A. Aviña-Verduzco[2*]

NMR Spectra……………………………………………..……………………….……S2

Docking interactions…………………..……………………………………………….S16

Per residue decomposition….…………………..………..………………….…………S18

i

NMR SPECTRA

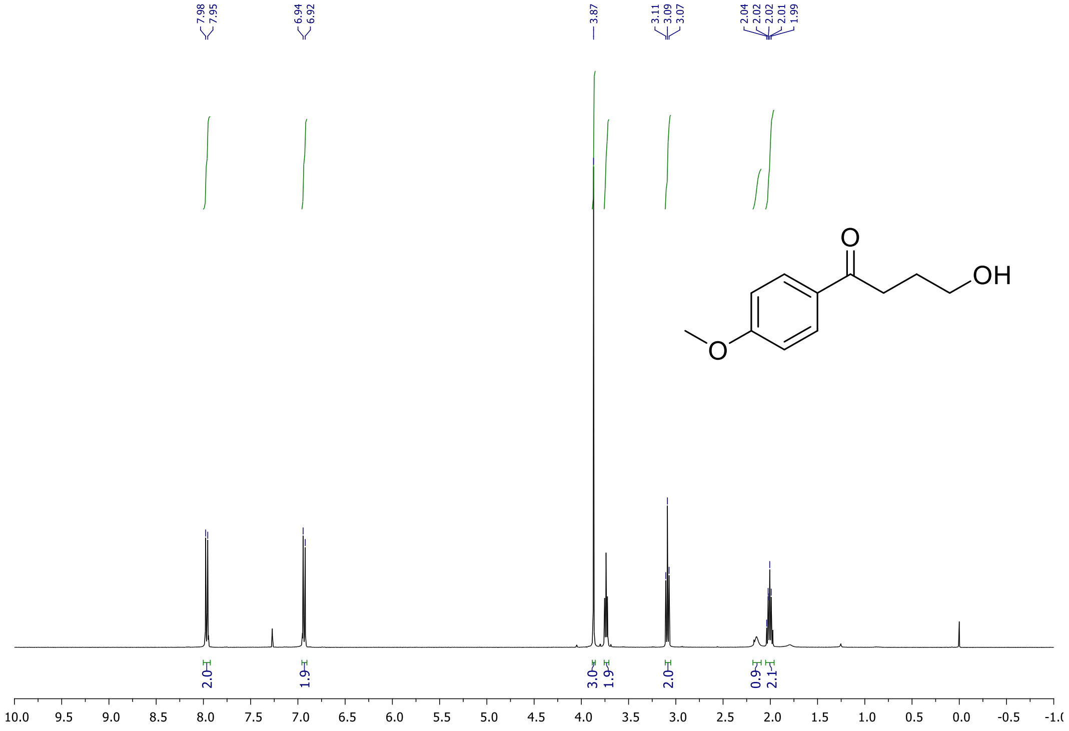

Figure S1. ^{1}H NMR spectrum of compound **1** (400 MHz, $CDCl_3$).

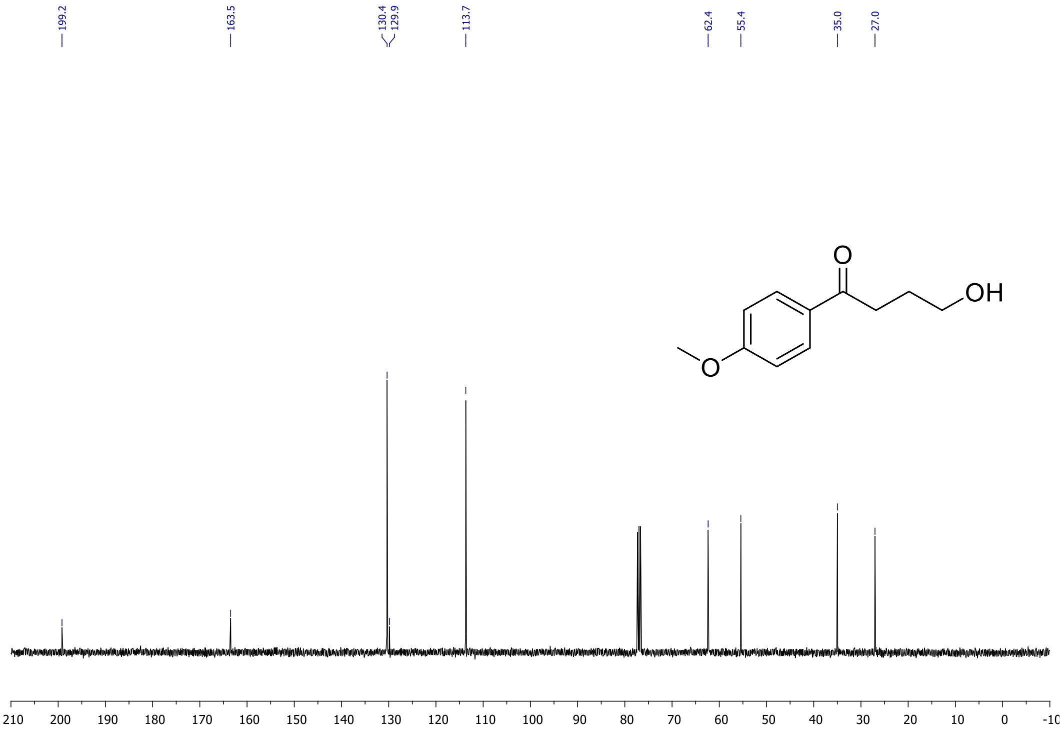

Figure S2. ^{13}C NMR spectrum of compound **1** (100 MHz, $CDCl_3$).

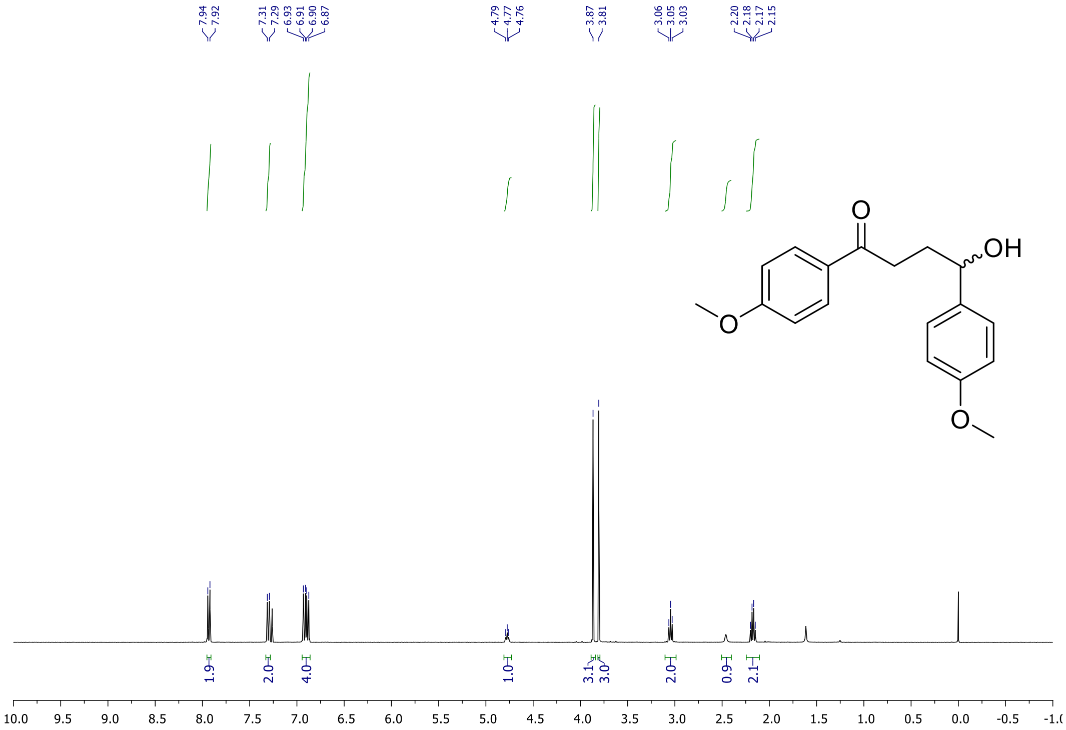

Figure S3. ^{1}H NMR spectrum of compound **2** (400 MHz, $CDCl_3$).

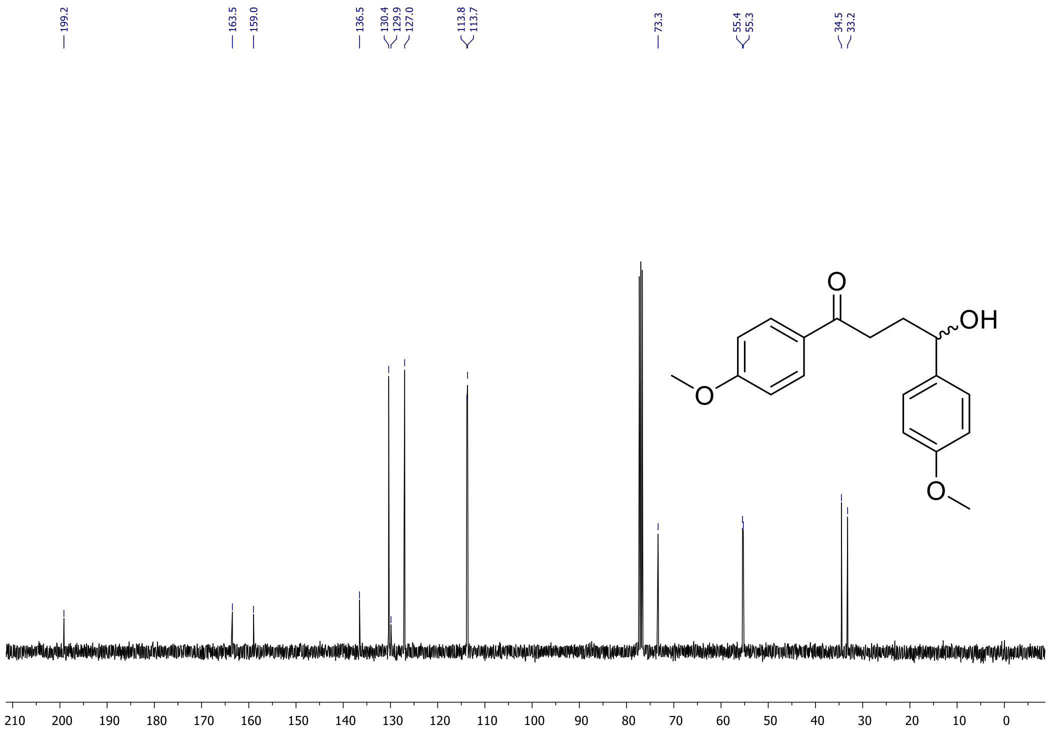

Figure S4. ^{13}C NMR spectrum of compound **2** (100 MHz, $CDCl_3$).

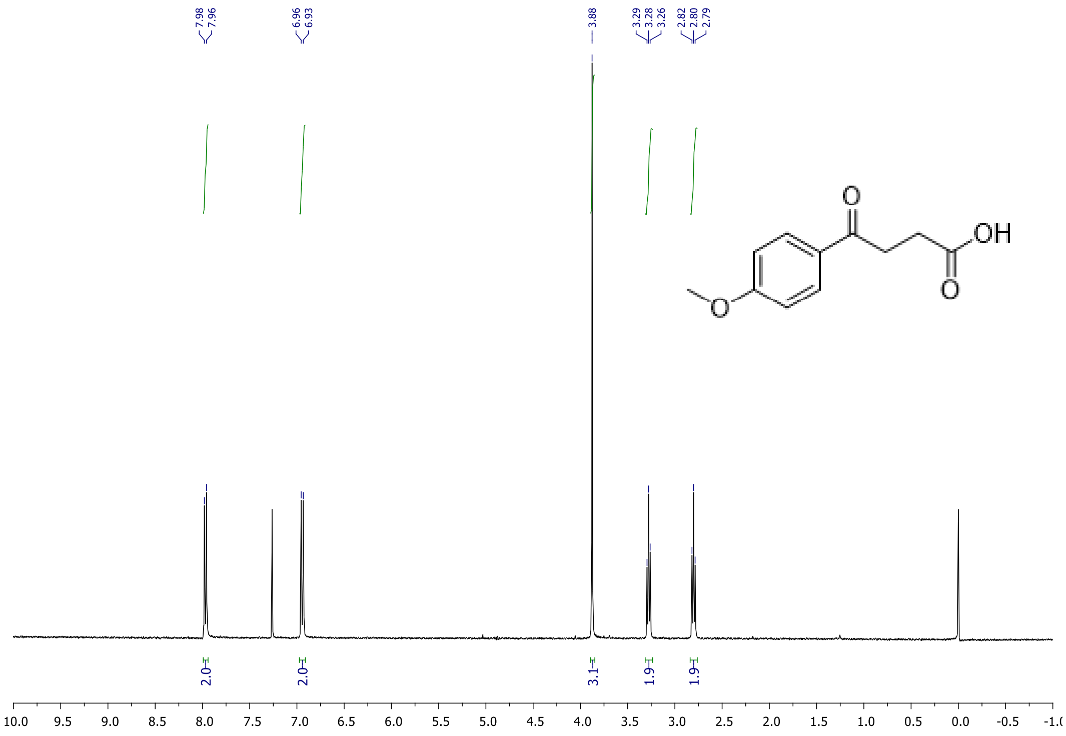

Figure S5. ^{1}H NMR spectrum of compound **3** (400 MHz, $CDCl_3$).

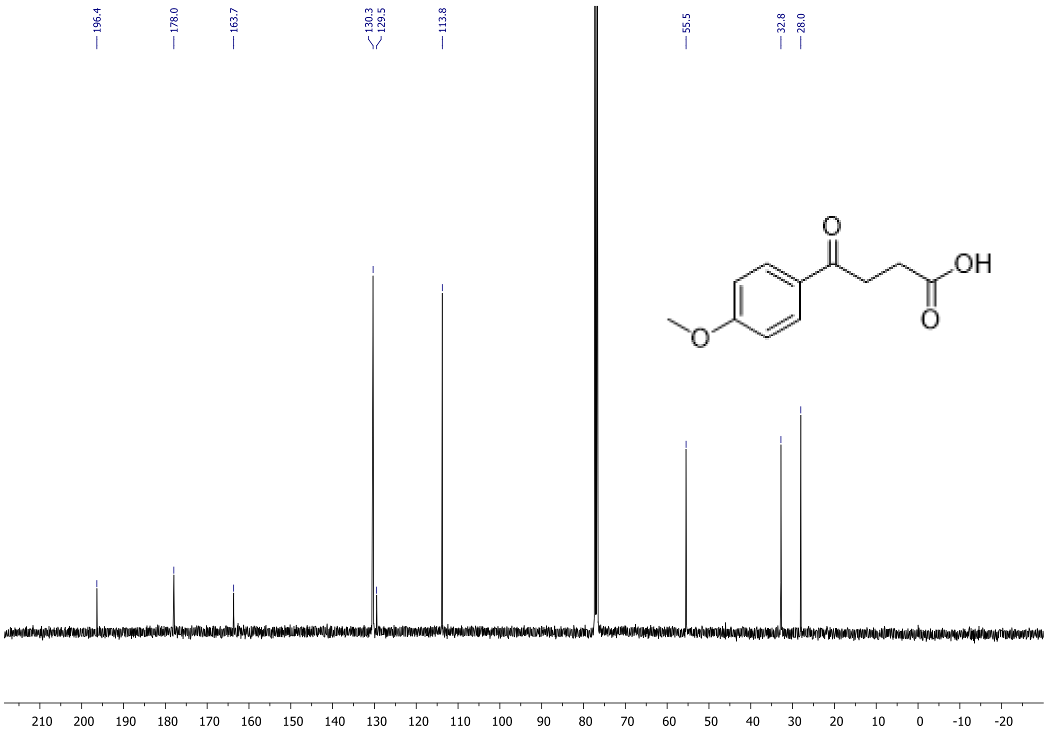

Figure S6. ^{13}C NMR spectrum of compound **3** (100 MHz, $CDCl_3$).

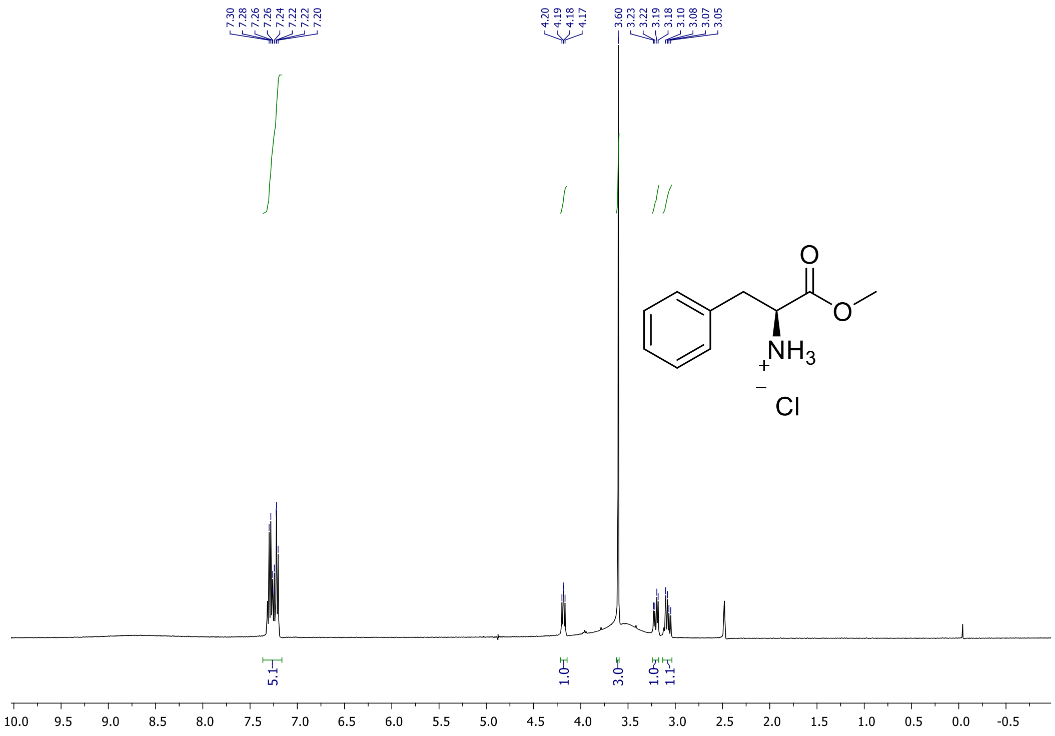

Figure S7. ^{1}H NMR spectrum of compound **4** (400 MHz, DMSO-d_6).

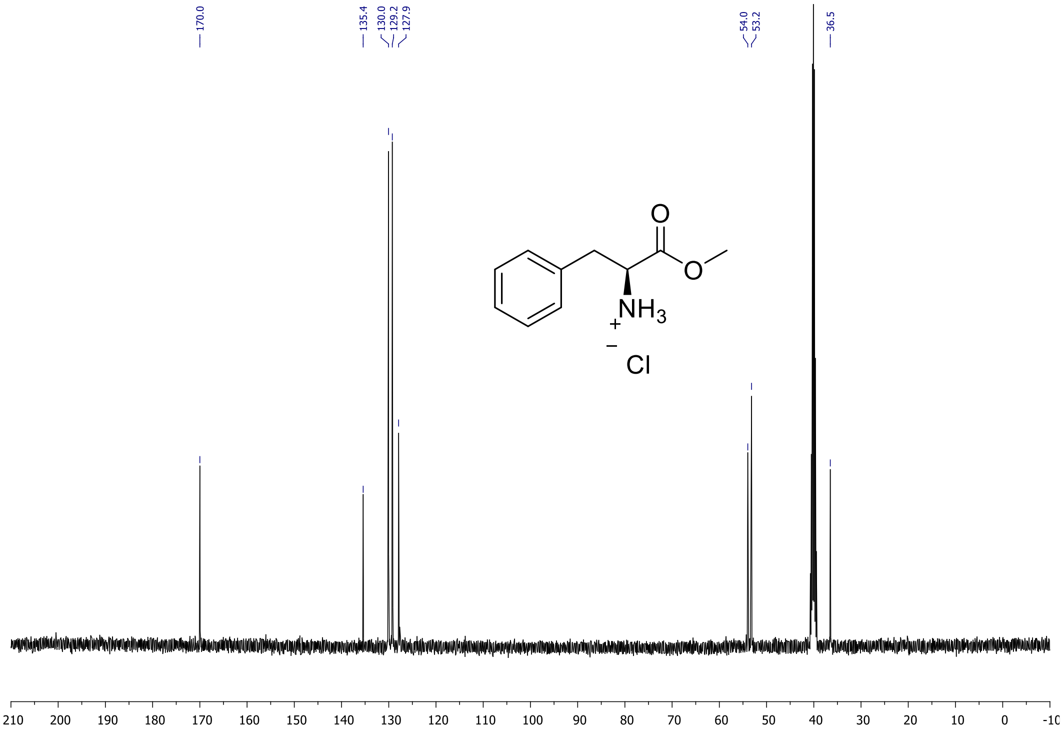

Figure S8. ^{13}C NMR spectrum of compound **4** (100 MHz, DMSO-d_6).

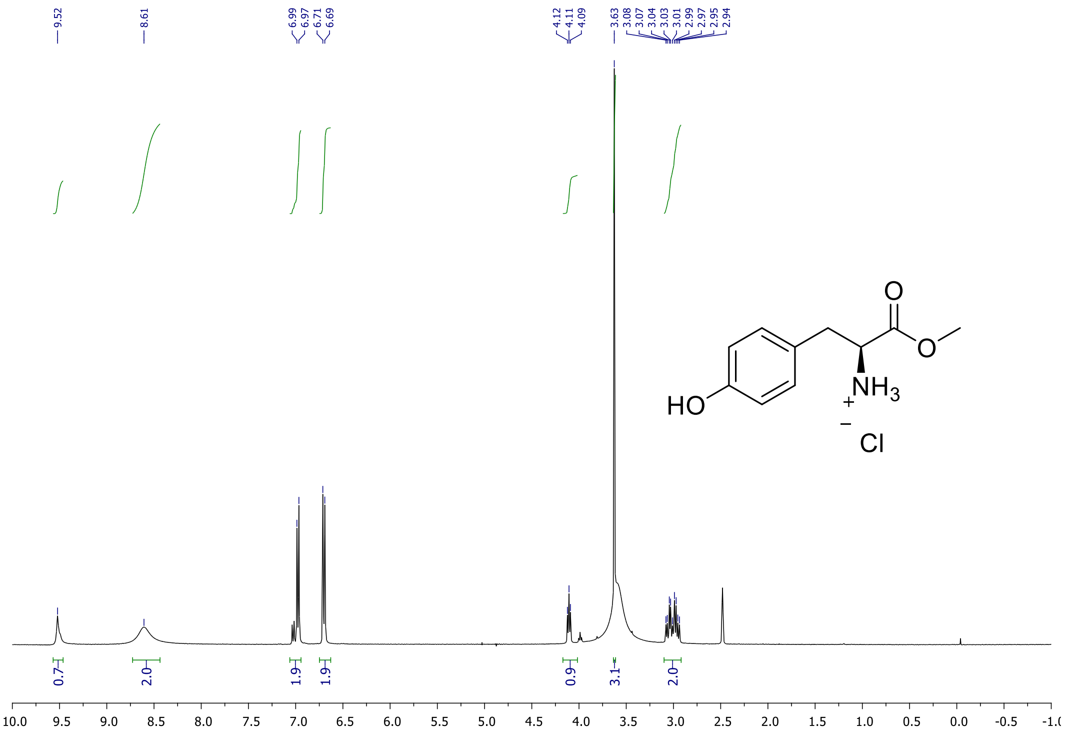

Figure S9. ^{1}H NMR spectrum of compound **5** (400 MHz, DMSO-d_6).

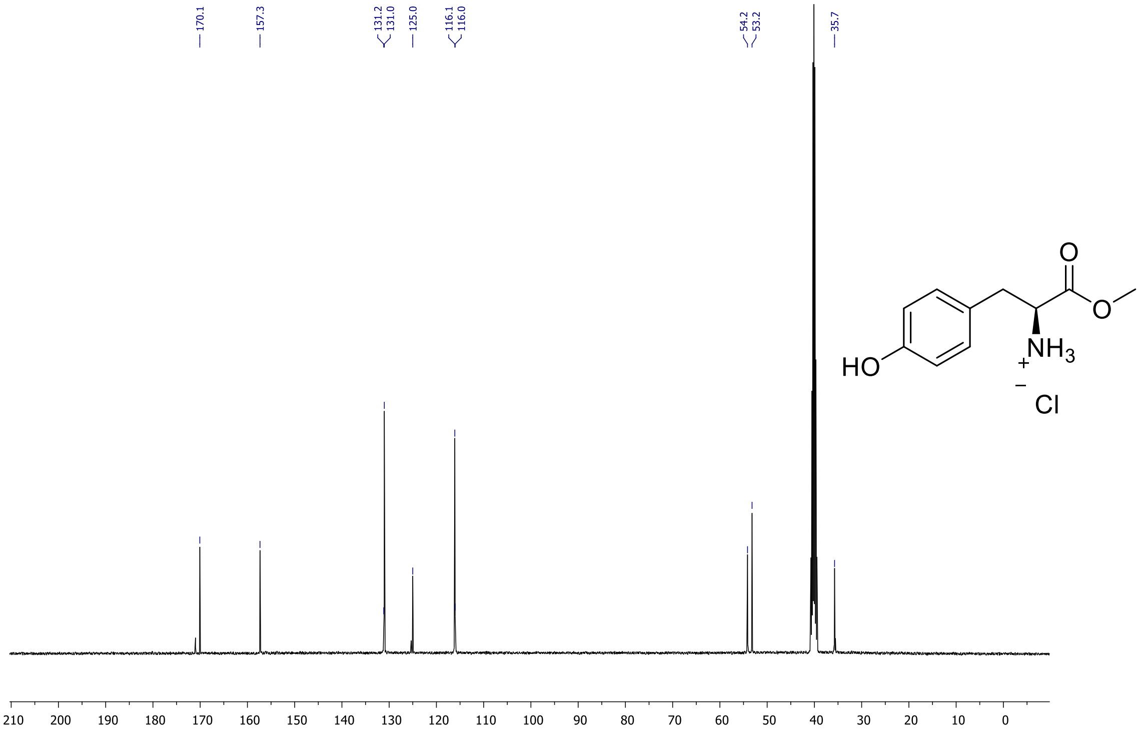

Figure S10. ^{13}C NMR spectrum of compound **5** (100 MHz, DMSO-d_6).

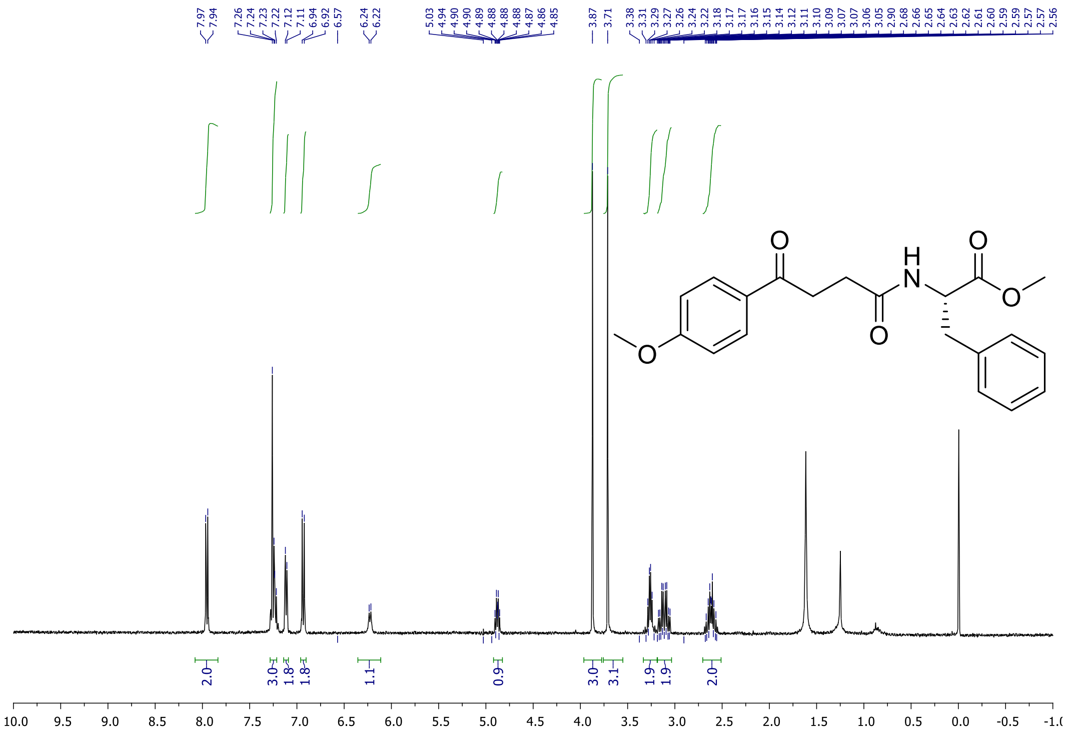

Figure S11. ^{1}H NMR spectrum of compound **6** (400 MHz, $CDCl_3$).

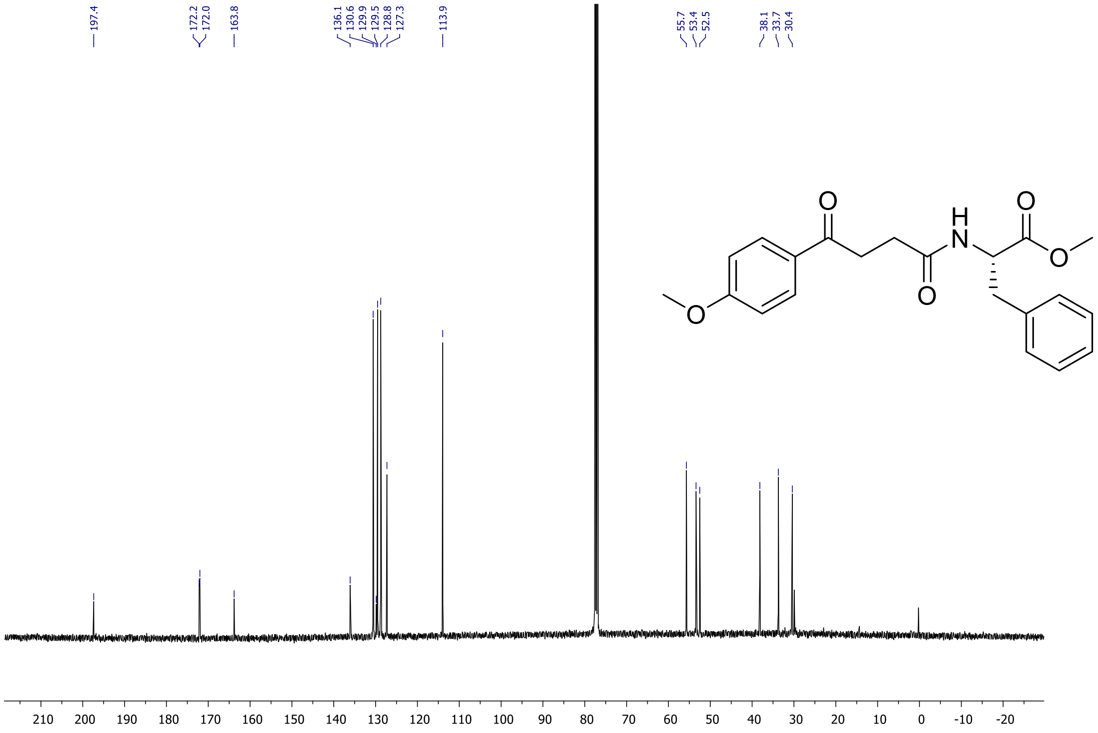

Figure S12. ^{13}C NMR spectrum of compound **6** (100 MHz, $CDCl_3$).

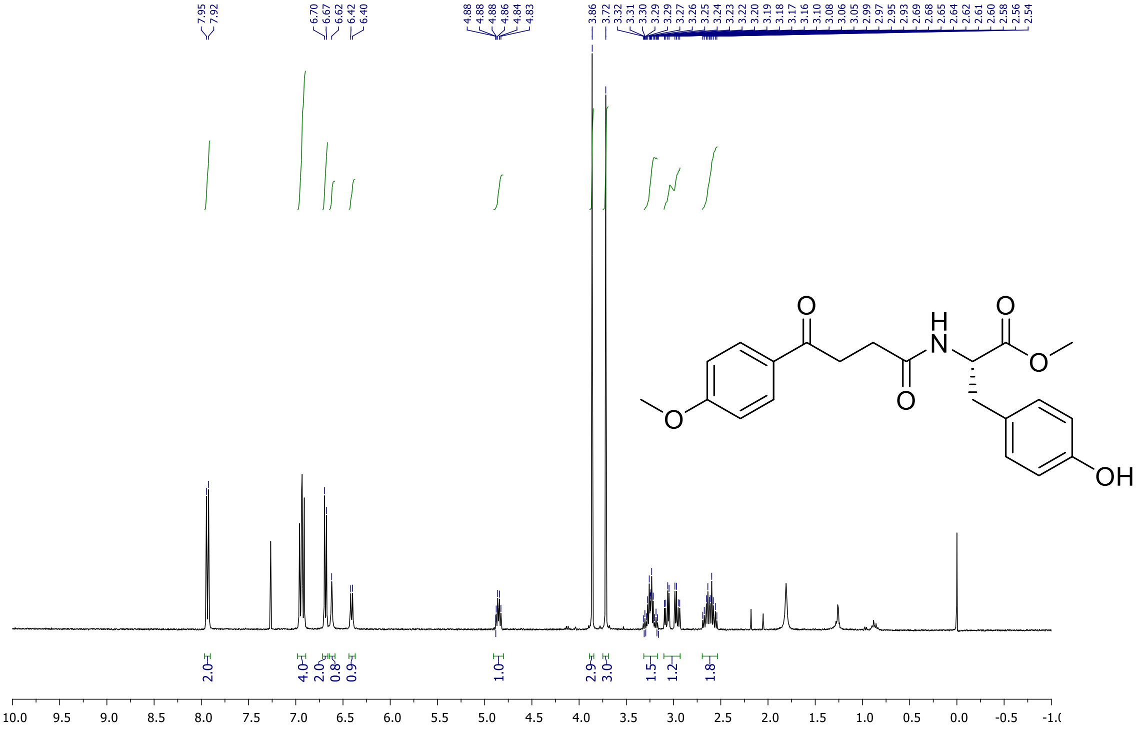

Figure S13. ^{1}H NMR spectrum of compound **7** (400 MHz, $CDCl_3$).

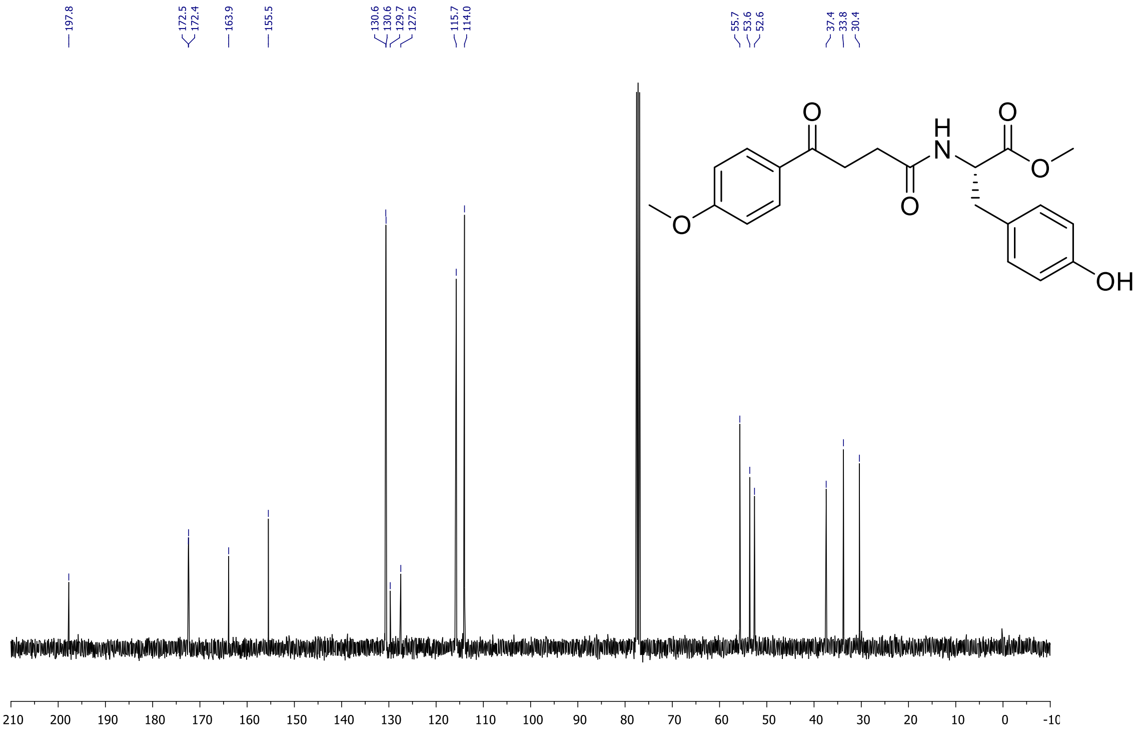

Figure S14. ^{13}C NMR spectrum of compound **7** (100 MHz, $CDCl_3$).

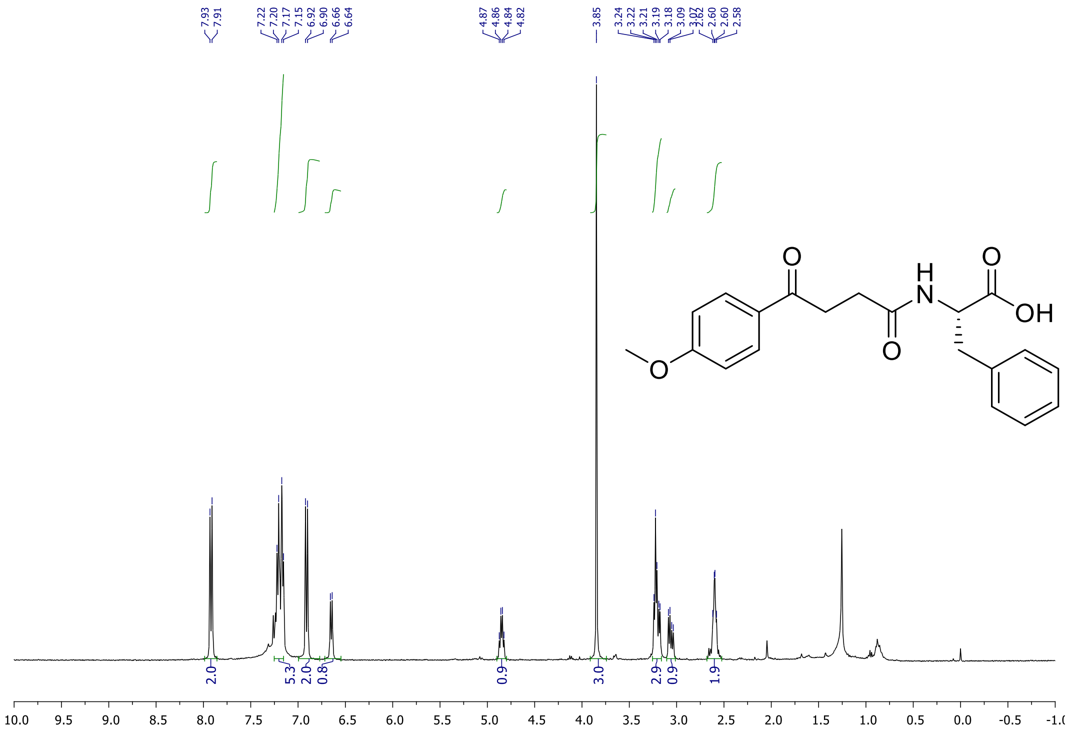

Figure S15. ^{1}H NMR spectrum of compound **8** (400 MHz, $CDCl_3$).

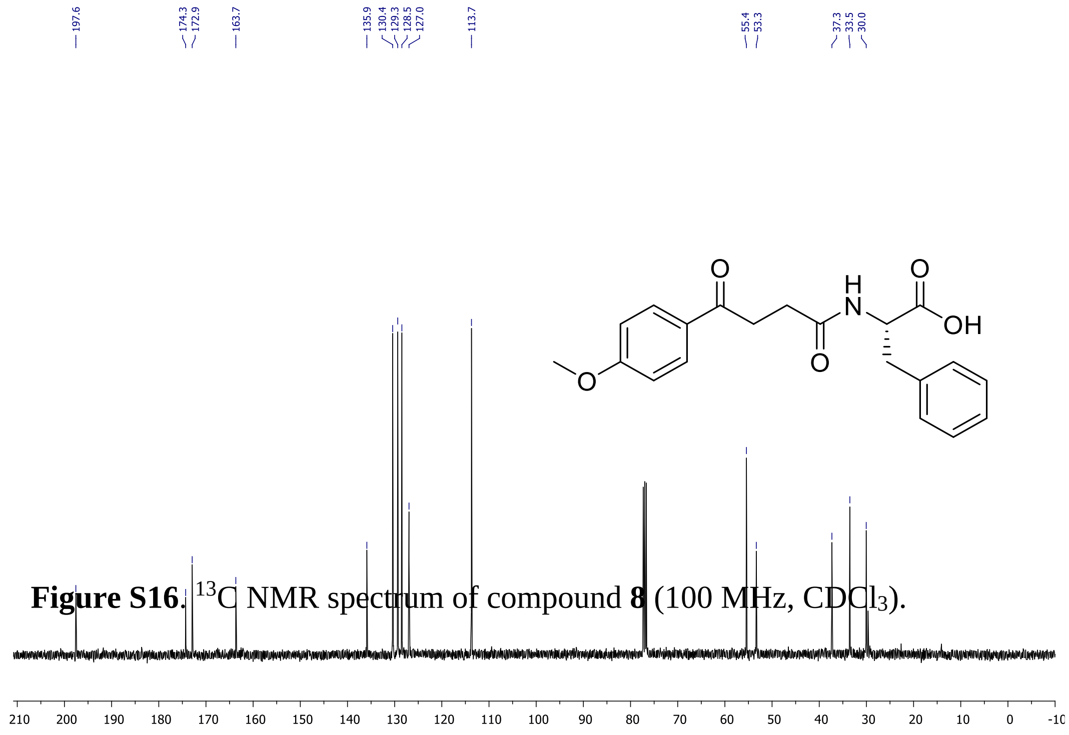

Figure S16. ^{13}C NMR spectrum of compound **8** (100 MHz, $CDCl_3$).

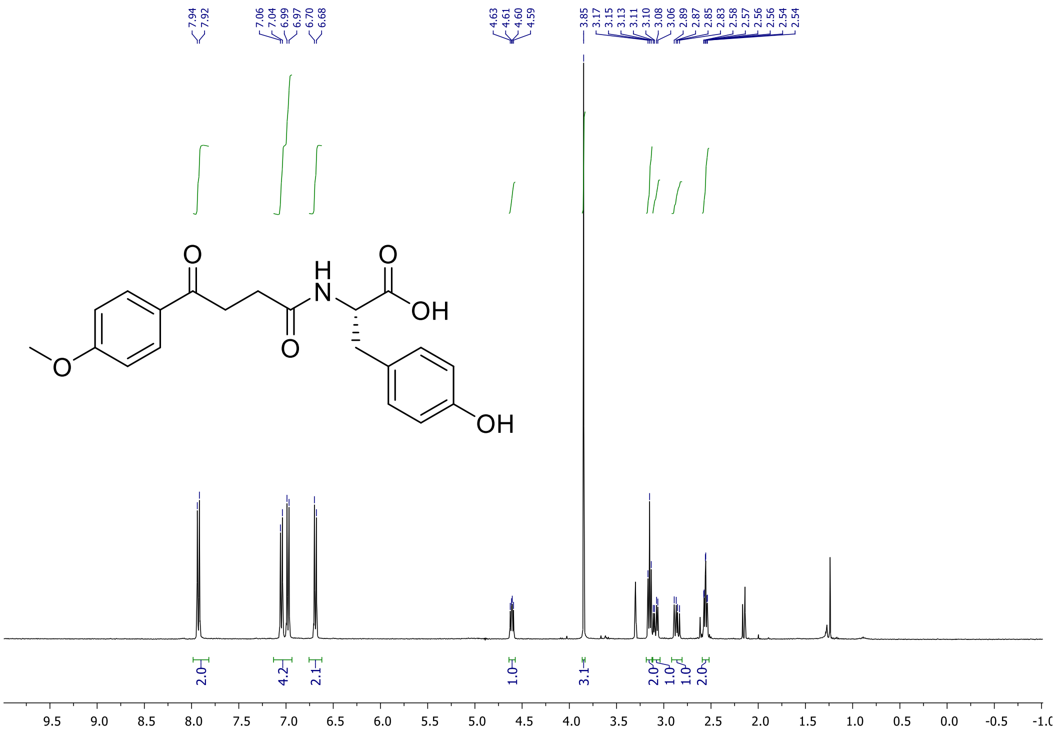

Figure S17. ^{1}H NMR spectrum of compound **9** (400 MHz, $CDCl_3$).

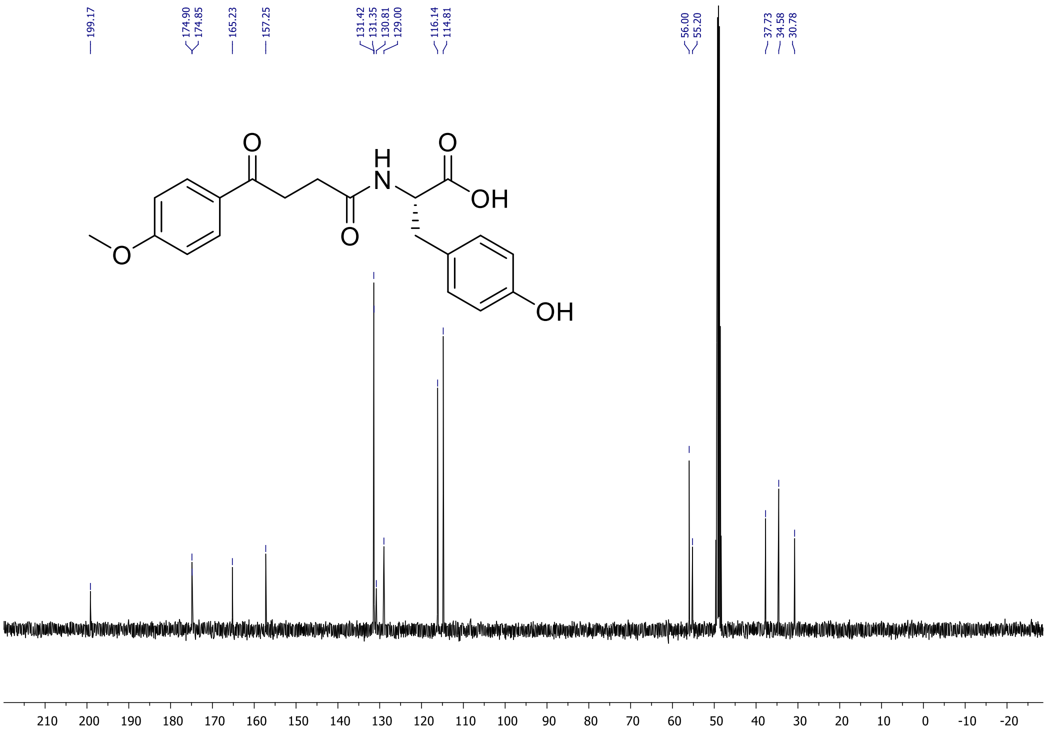

Figure S18. ^{13}C NMR spectrum of compound **9** (100 MHz, $CDCl_3$).

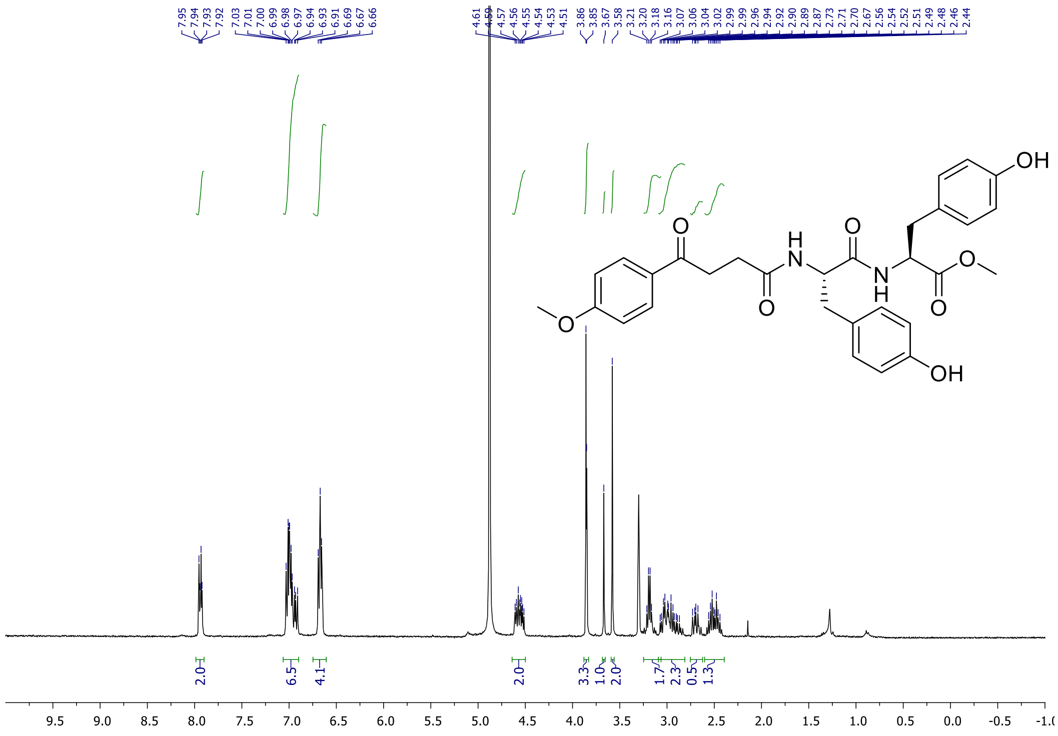

Figure S19. ^{1}H NMR spectrum of compound **10** (400 MHz, CD_3OD).

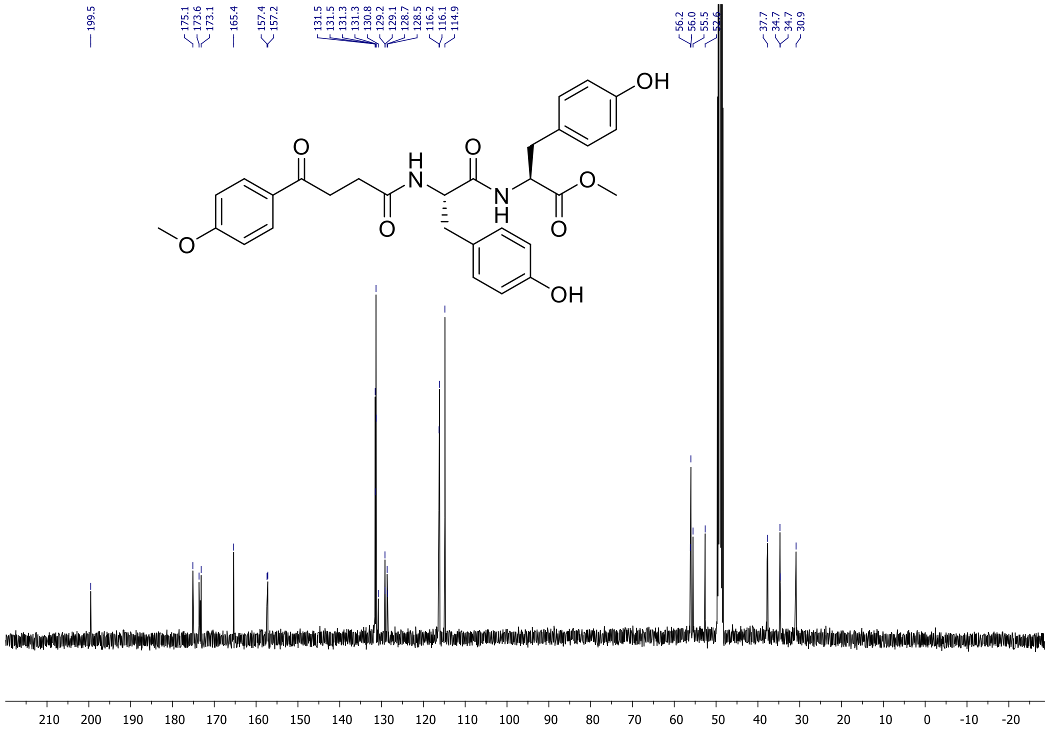

Figure S20. ^{13}C NMR spectrum of compound **10** (100 MHz, CD_3OD).

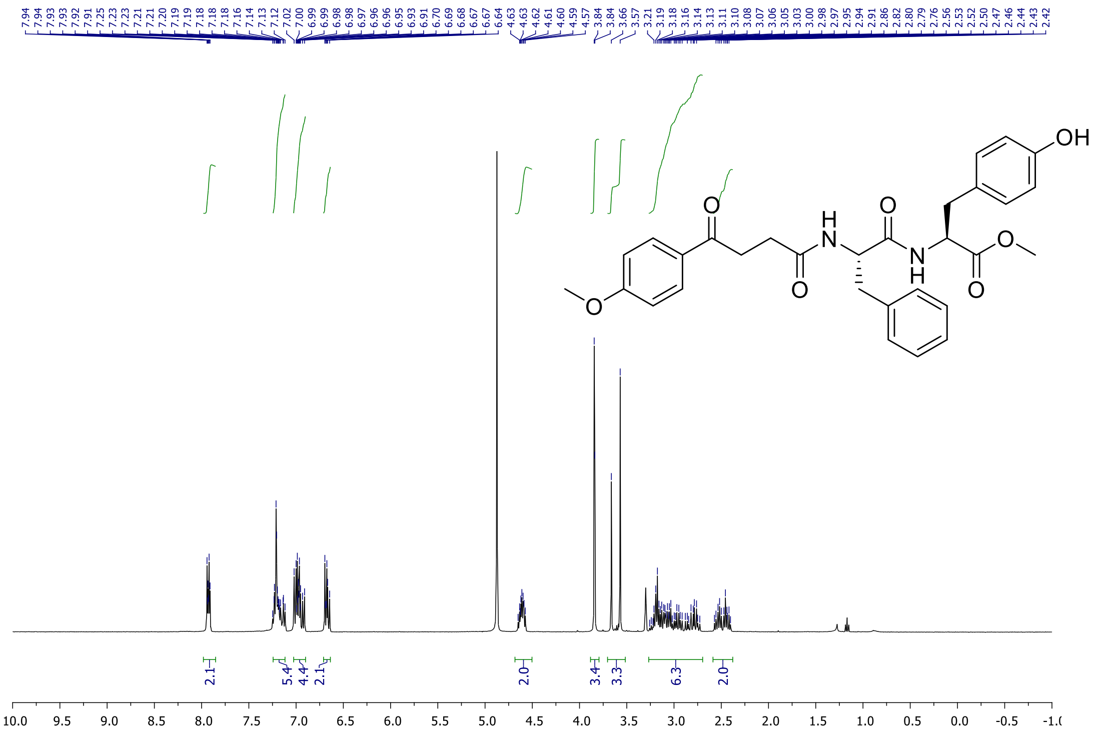

Figure S21. ^{1}H NMR spectrum of compound **11** (400 MHz, CD_3OD).

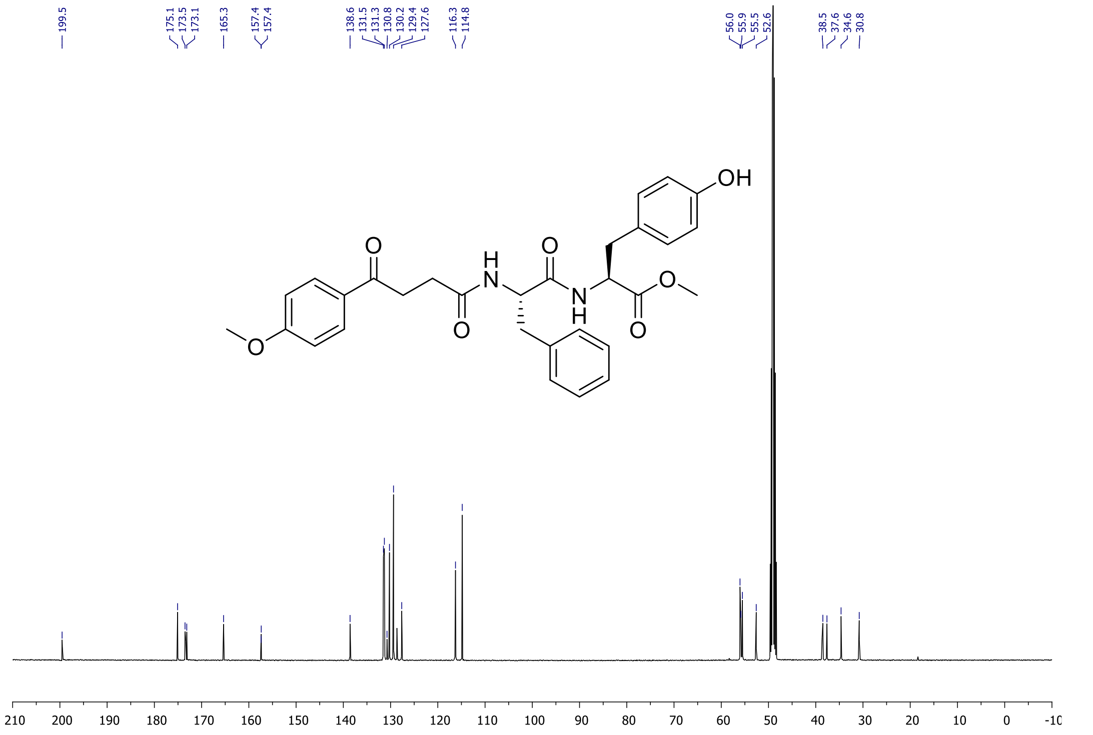

Figure S22. ^{13}C NMR spectrum of compound **11** (100 MHz, CD_3OD).

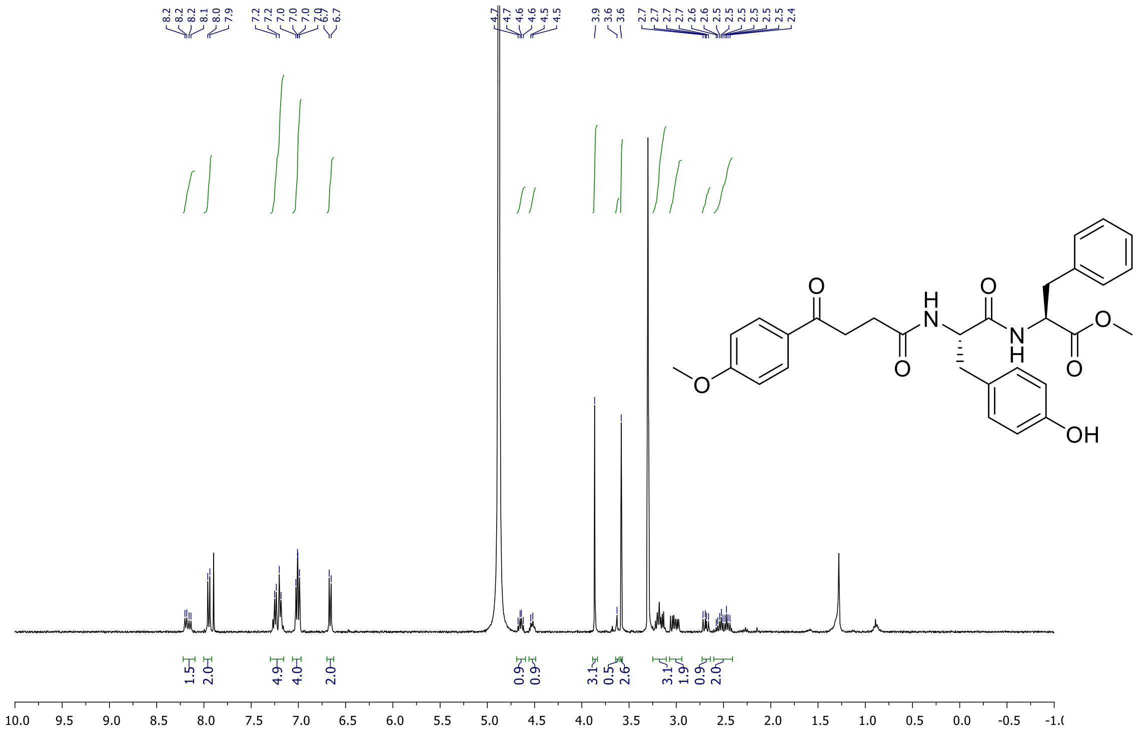

Figure S23. ^{1}H NMR spectrum of compound **12** (400 MHz, CD_3OD).

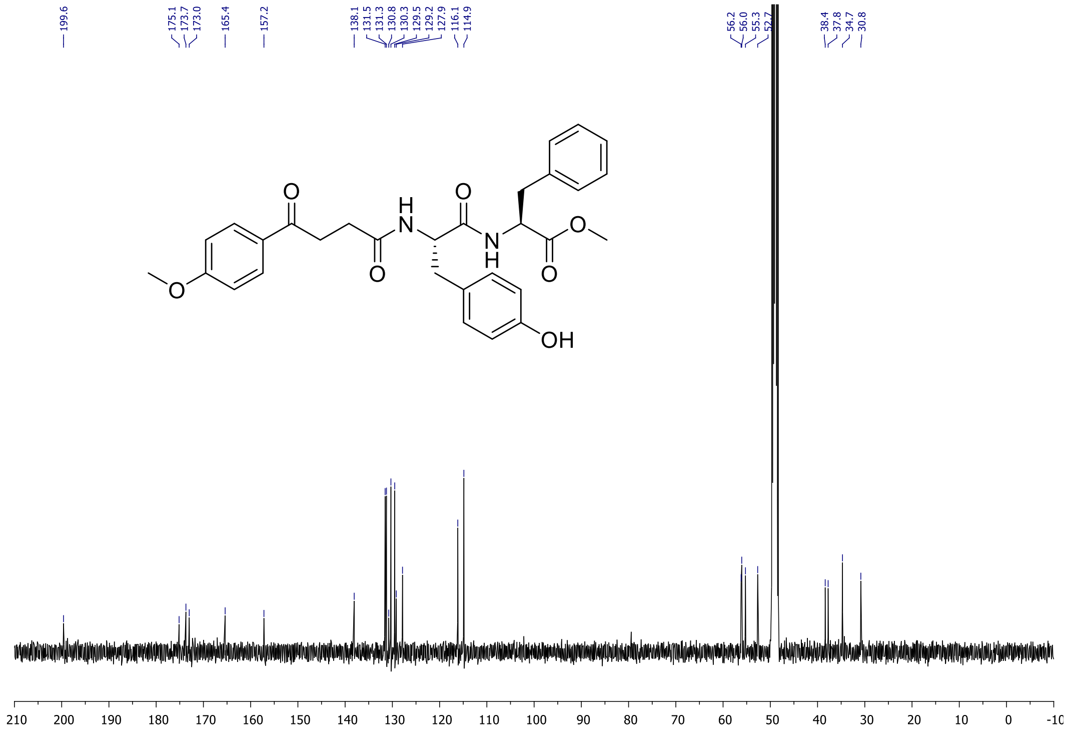

Figure S24. ^{13}C NMR spectrum of compound **12** (100 MHz, CD_3OD).

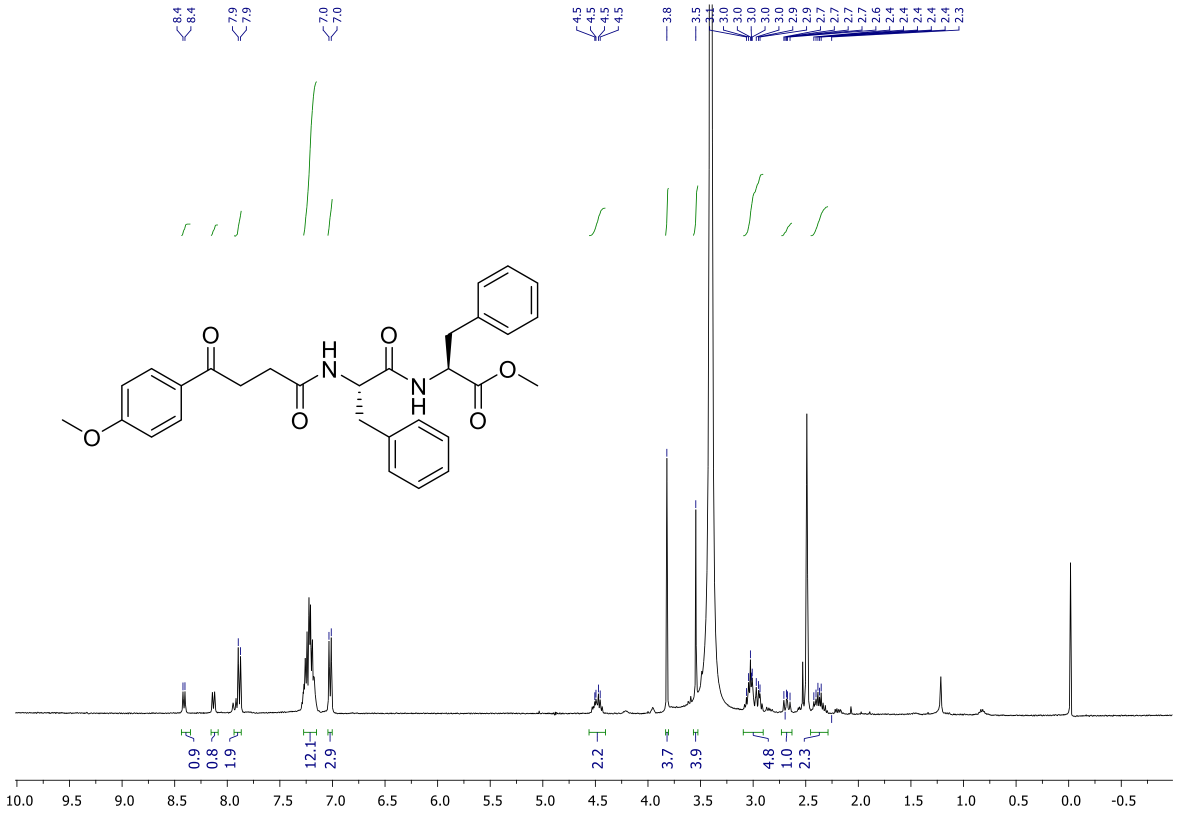

Figure S25. ^{1}H NMR spectrum of compound **13** (400 MHz, DMSO-d_6).

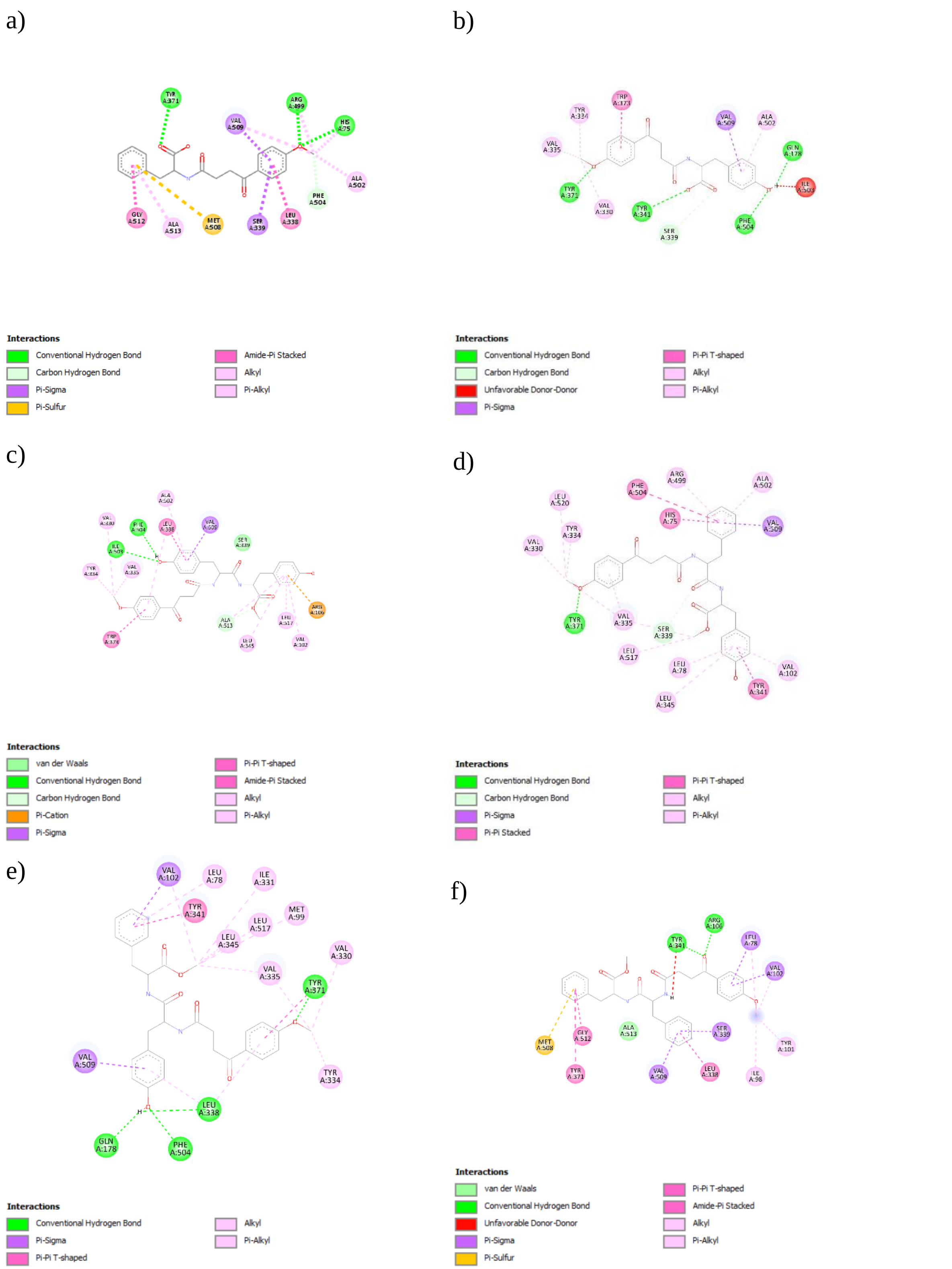

Figure S26. Observed interactions with protein 3LN1 and the ligands: a) **8**, b) **9**, c) **10**, d) **11**, e) **12** and f) **13**.

a)

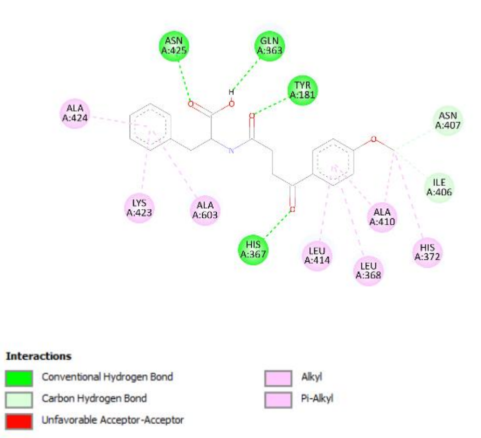

b)

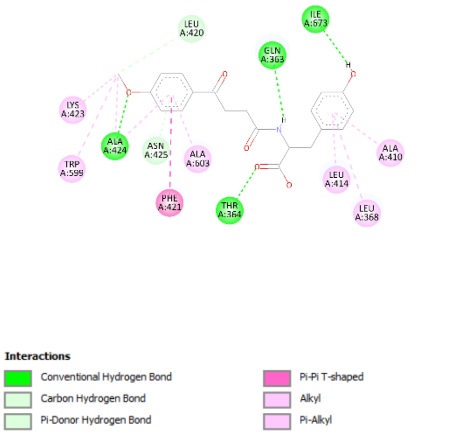

c)

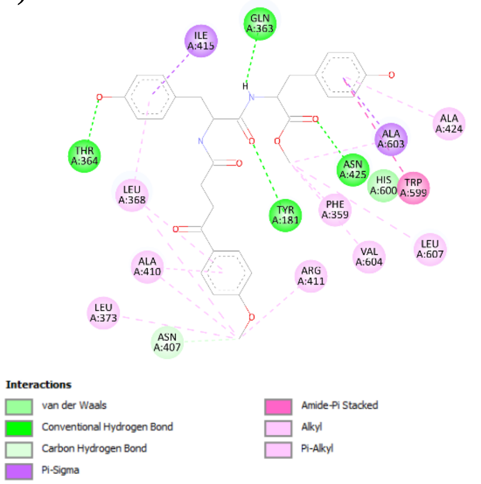

d)

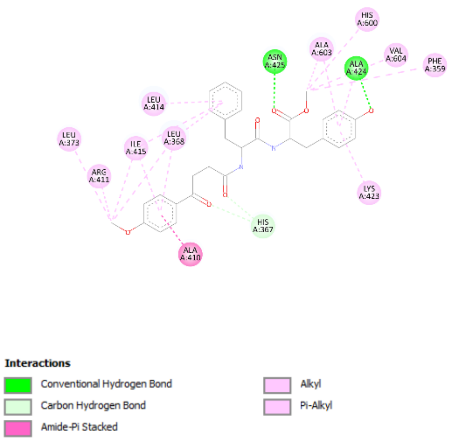

e)

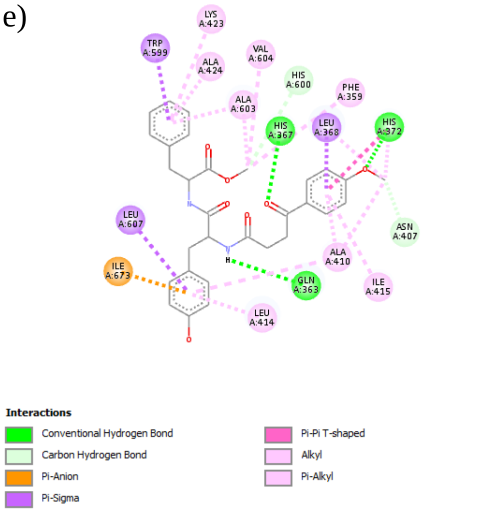

f)

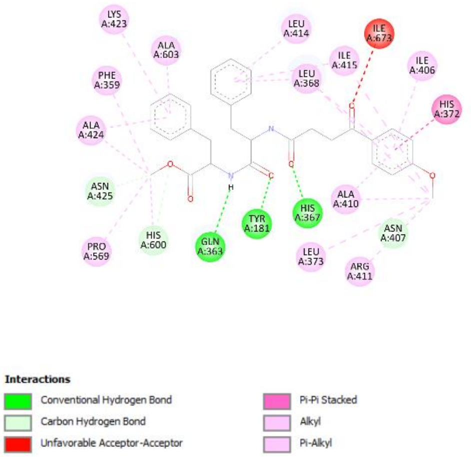

Figure S27. Observed interactions with protein 3O8Y and the ligands: a) **8**, b) **9**, c) **10**, d) **11**, e) **12** y f) **13**.

PER-RESIDUE ENERGY DECOMPOSITION

Table S1. Per-residue energy decomposition complex 3LN1 and compound **9**.

	Energy Decomposition Analysis (kcal/mol)			
Residue	van der Waals Avg.	Electrostatic Avg.	Polar Solvation Avg.	TOTAL Avg.
Pro71	-0.031	0.285	-0.014	0.239
Asn72	-0.011	0.282	-0.065	0.206
Val74	-0.035	-0.601	0.141	-0.495
His75	-0.206	-0.874	0.427	-0.653
Tyr76	-0.010	-0.106	0.137	0.020
Leu77	-0.070	-0.635	0.158	-0.547
Thr79	-0.040	-0.601	0.081	-0.561
Met99	-0.023	0.920	-0.265	0.632
Val102	-0.114	0.667	-0.212	0.340
Arg106	1.527	-55.255	16.586	-37.143
Asp176	-0.007	9.352	-7.879	1.467
Pro177	-0.004	-0.160	0.081	-0.083
Gln178	-0.011	-0.355	0.093	-0.274
Ser180	-0.008	-0.034	0.015	-0.027
Phe184	-0.068	-0.048	-0.006	-0.123
Phe187	-0.025	-0.006	0.001	-0.030
Ala188	-0.219	0.090	-0.036	-0.165
Phe191	-0.102	-0.119	0.078	-0.142
Thr192	-0.324	-0.123	0.080	-0.366
Phe195	-0.030	-0.140	0.052	-0.118
Phe196	-0.014	-0.042	0.064	0.007
Val230	-0.003	-0.179	0.034	-0.148
Thr326	-0.003	0.220	-0.088	0.129
ile327	-0.006	0.393	-0.123	0.264
ile329	-0.005	0.343	-0.159	0.179
Val330	-0.027	0.389	-0.118	0.243
Ile331	-0.032	0.477	-0.119	0.326
Asp333	-0.010	9.366	-8.151	1.205
Tyr334	-0.369	-0.142	0.233	-0.278
Val335	-0.554	0.339	-0.077	-0.292
Gln336	-0.077	0.882	-0.063	0.743
His337	-0.057	-0.297	-0.079	-0.434
Leu338	-1.887	0.079	-0.020	-1.828
Ser339	-1.271	-1.940	0.105	-3.106
Gly340	-0.118	-0.155	-0.002	-0.275
Tyr341	-0.989	-2.495	0.469	-3.015
Leu345	-0.109	-0.931	0.121	-0.920
Glu366	-0.009	9.919	-8.179	1.731
Phe367	-0.655	0.049	-0.011	-0.617
Asn368	-0.031	0.627	-0.170	0.427
Leu370	-0.334	-0.043	0.028	-0.349
Tyr371	-1.538	-1.205	0.073	-2.670
Trp373	-0.513	0.008	0.157	-0.348
Arg419	-0.012	-8.189	7.337	-0.863
Val420	-0.106	-0.316	0.105	-0.317
Ala421	-0.017	-0.344	0.114	-0.247
Leu493	-0.050	0.233	-0.059	0.124
Pro498	-0.033	-0.364	-0.094	-0.492
Arg499	-0.667	-20.402	11.766	-9.302
Pro500	-0.098	0.480	0.061	0.444
Asp501	-0.059	10.581	-9.529	0.992
Ala502	-0.289	-0.919	0.055	-1.153
Ile503	-0.189	-0.587	0.079	-0.697
Phe504	-2.017	-0.138	-0.001	-2.156
Gly505	-0.095	1.329	-0.162	1.072
Glu506	-0.076	14.430	-10.939	3.415
Thr507	-0.045	0.565	-0.185	0.335
Met508	-1.211	0.956	-0.115	-0.371
Val509	-2.616	0.658	-0.150	-2.108
Glu510	-0.292	27.919	-13.034	14.593
Leu511	-0.165	-0.046	-0.142	-0.353

Gly512	-0.765	-1.184	0.109	-1.840
Ala513	-1.378	-1.269	0.353	-2.294
Pro514	-0.152	0.137	0.108	0.093
Phe515	-0.094	-0.389	0.039	-0.444
Ser516	-0.683	-2.813	0.819	-2.677
Leu517	-0.287	-0.975	0.318	-0.944
Lys518	-0.020	-11.683	8.895	-2.808
Gly519	-0.012	-0.669	0.161	-0.520
Leu520	-0.107	-0.969	0.232	-0.843

Table S2. Per-residue energy decomposition complex 3LN1 and compound **12**.

	Energy Decomposition Analysis (kcal/mol)			
Residue	van der Waals Avg.	Electrostatic Avg.	Polar Solvation Avg.	TOTAL Avg.
Pro71	-0.048	-0.030	0.052	-0.026
Val74	-1.884	-2.851	0.774	-3.962
His75	-1.651	-1.957	0.032	-3.577
Tyr76	-1.900	-1.928	0.158	-3.671
Ile77	-0.092	0.122	0.020	0.051
Leu78	-0.071	0.060	-0.013	-0.024
Thr79	-0.007	-0.028	0.020	-0.016
Trp85	-0.013	-0.014	0.000	-0.027
Arg95	-0.012	0.295	-0.289	-0.006
Ile98	-0.039	-0.002	-0.007	-0.048
Met99	-0.575	0.017	-0.010	-0.568
Lys100	-0.036	-0.112	0.114	-0.034
Tyr101	-0.066	0.052	-0.047	-0.061
Val102	-1.208	0.143	-0.009	-1.074
Leu103	-0.951	0.167	-0.072	-0.856
Thr104	-0.079	-0.002	-0.001	-0.082
Ser105	-0.261	-0.114	0.046	-0.328
Arg106	-0.678	0.647	0.216	0.185
Asp176	-0.003	0.113	0.186	0.296
Pro177	-0.002	-0.008	-0.002	-0.012
Gln178	-0.005	-0.023	-0.020	-0.048
Ser180	-0.005	0.008	-0.004	-0.002
Phe184	-0.012	-0.003	0.016	0.000
Phe187	-0.019	0.041	-0.020	0.002
Ala188	-0.033	0.059	-0.026	0.000
Phe191	-0.071	-0.026	-0.046	-0.143
Thr192	-0.144	-0.065	-0.055	-0.263
Phe195	-0.071	-0.006	-0.043	-0.120
Phe196	-0.285	0.028	-0.018	-0.274
Val214	-0.006	-0.003	-0.013	-0.021
Thr326	-0.008	-0.028	0.053	0.016
Ile327	-0.021	-0.041	0.072	0.010
Ile329	-0.024	-0.066	0.077	-0.012
Val330	-0.165	-0.127	0.193	-0.099
Ile331	-0.751	-0.358	0.293	-0.816
Glu332	-0.103	-0.561	0.606	-0.057
Asp333	-0.033	-0.368	0.503	0.102
Tyr334	-1.096	-2.118	0.311	-2.904
Val335	-1.597	0.104	-0.109	-1.602
Gln336	-0.107	-0.041	0.017	-0.131
Hsd337	-0.029	-0.003	-0.063	-0.095
Leu338	-0.294	-0.052	-0.089	-0.435
Ser339	-0.190	0.004	-0.087	-0.273
Gly340	-0.016	0.028	-0.016	-0.004
Tyr341	-0.234	0.037	-0.030	-0.228
His342	-0.009	0.053	-0.030	0.014
Phe343	-0.096	-0.056	0.031	-0.122
Lys344	-0.024	0.499	-0.488	-0.013
Leu345	-0.651	-0.006	0.041	-0.616
Lys346	-0.039	0.317	-0.346	-0.068
Phe347	-0.047	0.012	-0.025	-0.060
Asp348	-0.014	-0.071	0.108	0.023
Phe367	-0.695	0.047	0.065	-0.584
Leu370	-0.033	0.024	0.018	0.010

Tyr371	-2.019	-0.424	0.162	-2.280
Trp373	-0.351	0.061	0.017	-0.273
Leu376	-0.011	-0.007	-0.011	-0.029
Arg419	-0.009	-0.102	-0.131	-0.242
Val420	-0.022	-0.025	-0.023	-0.070
Ala421	-0.004	0.001	-0.006	-0.010
Leu493	-0.013	0.006	0.006	0.000
Pro498	-0.006	-0.007	-0.014	-0.027
Arg499	-0.007	0.261	-0.225	0.029
Pro500	-0.003	-0.025	0.029	0.001
Asp501	-0.007	-0.231	0.289	0.051
Ala502	-0.014	-0.028	-0.034	-0.077
Ile503	-0.062	-0.090	-0.031	-0.183
Phe504	-0.637	-0.058	0.034	-0.661
Gly505	-0.018	-0.009	0.020	-0.007
Glu506	-0.026	-0.415	0.289	-0.152
Thr507	-0.021	-0.035	-0.010	-0.066
Met508	-0.233	-0.189	0.002	-0.420
Val509	-0.945	-0.080	-0.063	-1.089
Glu510	-0.143	-0.609	0.072	-0.679
Leu511	-0.133	0.158	-0.058	-0.032
Gly512	-1.018	1.215	-0.151	0.045
Ala513	-1.483	1.350	-0.245	-0.378
Pro514	-0.316	0.276	-0.061	-0.101
Phe515	-0.232	-0.105	-0.030	-0.367
Ser516	-1.532	-3.707	1.187	-4.052
Leu517	-1.433	-0.786	0.151	-2.067
Lys518	-0.068	-1.238	0.608	-0.698
Gly519	-0.021	-0.073	0.049	-0.045
leu520	-0.107	-0.188	0.045	-0.250
Met421	-0.070	-0.167	0.023	-0.214
HEM570	-1.759	-0.641	0.396	-2.004

Table S3. Per-residue energy decomposition complex 3O8Y and compound **8**.

	Energy Decomposition Analysis (kcal/mol)			
Residue	van der Waals Avg.	Electrostatic Avg.	Polar Solvation Avg.	TOTAL Avg.
Trp147	-0.017	0.045	-0.088	-0.060
Phe151	-0.008	-0.282	0.097	-0.193
Phe169	-0.016	-0.215	0.136	-0.095
Val175	-0.015	0.179	0.011	0.175
Phe177	-0.906	0.001	-0.008	-0.913
Val178	-0.045	0.160	0.035	0.150
Asn180	-0.047	-0.325	-0.035	-0.406
Tyr181	-0.919	-2.899	0.158	-3.660
Ser181	-0.011	-0.017	0.008	-0.020
Ala184	-0.012	-0.372	0.073	-0.312
Met185	-0.039	-0.152	0.063	-0.128
Met194	-0.014	0.288	-0.093	0.182
Phe197	-0.020	0.073	0.027	0.080
Asn295	-0.016	-1.444	0.053	-1.407
Gln303	-0.010	0.491	-0.098	0.382
Phe359	-0.432	0.148	-0.027	-0.311
His360	-0.137	-2.160	0.085	-2.211
Val361	-0.022	0.266	-0.002	0.243
His362	-0.081	-1.117	0.053	-1.145
Gln363	-1.903	-9.242	0.818	-10.327
Thr364	-0.095	-1.518	0.127	-1.486
Ile365	-0.023	-0.335	0.096	-0.262
Thr366	-0.022	-0.627	0.043	-0.606
His367	-1.180	-1.386	0.626	-1.941
Leu368	-0.516	-0.535	0.083	-0.968
Leu369	-0.024	-0.356	0.105	-0.275
Arg370	-0.010	-9.247	7.359	-1.898
Thr371	-0.027	-0.207	0.065	-0.168
His372	-0.927	-0.068	-0.010	-1.006
Leu373	-0.075	-0.250	0.097	-0.229
Ser375	-0.007	-0.352	0.132	-0.227

Glu376	-0.016	7.919	-6.272	1.630
Phe402	-0.013	0.248	-0.085	0.150
Thr403	-0.031	0.167	-0.051	0.085
Ile404	-0.015	0.234	-0.095	0.124
Ala405	-0.019	0.137	-0.061	0.057
Ile406	-0.749	0.132	-0.044	-0.661
Asn407	-0.426	0.786	-0.198	0.161
Thr408	-0.034	0.005	-0.056	-0.086
Lys409	-0.072	-8.447	7.200	-1.319
Ala410	-0.445	-0.035	-0.116	-0.596
Arg411	-0.070	-8.274	6.518	-1.826
Glu412	-0.015	7.931	-7.264	0.651
Gln413	-0.050	0.391	-0.174	0.166
Leu414	-1.653	0.257	-0.171	-1.567
Ile415	-0.316	-0.068	-0.067	-0.451
Cys416	-0.023	0.675	-0.096	0.555
Gly419	-0.041	0.117	-0.078	-0.002
Leu420	-0.928	0.913	-0.106	-0.121
Phe421	-1.698	0.121	-0.034	-1.610
Asp422	-0.084	13.671	-9.104	4.482
Lys423	-0.123	-11.480	9.773	-1.830
Ala424	-0.809	-0.576	0.059	-1.325
Asn425	-0.446	-10.058	0.508	-9.997
Ala426	-0.044	-1.172	-0.004	-1.220
Thr427	-0.041	-1.397	0.083	-1.355
Gly428	-0.022	-0.718	0.041	-0.698
Gly429	-0.007	0.388	0.108	0.489
His432	-0.050	-0.156	0.228	0.022
Val436	-0.009	-0.271	0.065	-0.215
His450	-0.057	1.128	-0.255	0.815
Val553	-0.031	0.581	-0.084	0.466
Asn554	-0.102	1.007	-0.130	0.775
Gln557	-0.240	1.777	-0.121	1.416
Ala561	-0.027	-0.214	0.007	-0.234
Ala567	-0.050	1.552	-0.091	1.411
Pro568	-0.088	1.042	-0.032	0.921
Pro569	-0.807	-0.719	0.045	-1.481
Thr570	-0.065	0.395	0.044	0.373
Gly595	-0.018	0.430	0.003	0.415
Arg596	-0.160	-13.492	9.069	-4.583
Ser597	-0.052	1.039	-0.042	0.945
Cys598	-0.051	0.252	0.031	0.232
Trp599	-1.048	-0.195	0.036	-1.206
His600	-0.745	-9.166	0.530	-9.380
Leu601	-0.098	0.310	-0.028	0.184
Gly602	-0.087	-0.189	0.017	-0.259
Ala603	-1.127	-0.870	0.072	-1.925
Val604	-0.291	-0.759	0.038	-1.012
Trp605	-0.042	-0.290	0.033	-0.300
Ala606	-0.100	-0.798	0.005	-0.893
Leu607	-1.159	-0.833	0.057	-1.934
Ser608	-0.018	-0.657	-0.015	-0.691
Val671	-0.055	-0.242	0.059	-0.238
Ala672	-0.052	-0.018	-0.065	-0.135
Ile673	-0.995	13.446	-7.482	4.969
Fe^{+2}674	-0.031	-27.684	15.231	-12.484

Table S4. Per-residue energy decomposition complex 3O8Y and compound **9**.

	Energy Decomposition Analysis (kcal/mol)			
Residue	van der Waals Avg.	Electrostatic Avg.	Polar Solvation Avg.	TOTAL Avg.
Trp147	-0.025	-0.014	-0.095	-0.135
Trp151	-0.014	-0.688	0.108	-0.594
Phe169	-0.014	-0.256	0.134	-0.136
Val175	-0.015	0.447	-0.081	0.351
Phe177	-0.932	-0.373	0.045	-1.259
Val178	-0.072	0.041	0.061	0.030

Asn180	-0.033	-0.756	0.163	-0.626
Tyr181	-1.474	-0.765	0.379	-1.860
Met185	-0.051	-0.116	0.075	-0.092
Met194	-0.021	0.145	-0.076	0.047
Phe197	-0.050	-0.007	-0.004	-0.061
Asn295	-0.014	-0.787	0.046	-0.755
Phe359	-0.562	1.436	-0.299	0.575
His360	-0.089	1.162	-0.094	0.979
Val361	-0.029	0.977	-0.144	0.804
His362	-0.132	0.005	0.022	-0.104
Gln363	-2.019	-10.764	1.774	-11.009
Thr364	0.036	-9.061	0.639	-8.386
Ile365	-0.090	0.811	-0.036	0.685
Thr366	-0.047	-0.852	0.019	-0.881
His367	-0.866	-14.037	1.828	-13.074
Leu368	-1.292	-5.743	0.531	-6.504
Leu369	-0.058	-0.801	0.120	-0.739
Arg370	-0.019	-13.434	8.840	-4.612
Thr371	-0.039	-0.908	0.126	-0.821
His372	-0.786	-1.301	0.001	-2.085
Leu373	-0.061	-0.835	0.164	-0.733
Thr403	-0.028	0.365	-0.078	0.259
Ala405	-0.015	0.237	-0.074	0.149
Ile406	-0.552	0.231	-0.034	-0.355
Asn407	-0.251	1.505	-0.309	0.945
Thr408	-0.025	-0.005	-0.067	-0.097
Lys409	-0.059	-10.115	8.188	-1.987
Ala410	-0.477	-0.713	0.037	-1.152
Arg411	-0.060	-12.955	8.212	-4.803
Glu412	-0.015	9.359	-8.354	0.990
Gln413	-0.046	-0.167	0.012	-0.202
Leu414	-1.378	-0.579	0.105	-1.853
Ile415	-0.597	-0.425	-0.002	-1.025
Cys416	-0.023	0.438	0.063	0.478
Gly419	-0.042	-0.341	0.098	-0.284
Leu420	-1.028	-0.177	0.119	-1.086
Phe421	-1.105	-0.169	0.100	-1.174
Asp422	-0.066	12.616	-8.780	3.770
Lys423	-0.227	-8.781	7.984	-1.024
Ala424	-0.735	-0.584	-0.071	-1.389
Asn425	-0.956	-1.737	0.795	-1.898
Ala426	-0.023	-0.296	-0.038	-0.357
Thr427	-0.041	-0.483	0.027	-0.497
Gly428	-0.020	-0.309	-0.018	-0.347
Gly429	-0.007	0.427	0.008	0.428
Gly431	-0.004	-0.027	-0.040	-0.070
His432	-0.047	-0.098	-0.036	-0.181
Val433	-0.006	0.217	0.054	0.265
Met435	-0.017	-0.562	0.037	-0.542
Val436	-0.011	-0.407	0.061	-0.358
His450	-0.070	2.041	-0.466	1.505
Val553	-0.040	0.329	-0.050	0.240
Asn554	-0.138	1.435	-0.306	0.991
Gln457	-0.102	-0.949	0.270	-0.781
Ala561	-0.019	-0.191	0.074	-0.135
Ile564	-0.018	0.227	-0.043	0.166
Ala567	-0.020	0.112	-0.006	0.086
Pro569	-0.350	0.100	-0.026	-0.276
Thr570	-0.032	-0.010	0.021	-0.021
Pro592	-0.016	-0.297	0.098	-0.216
Gly595	-0.033	0.264	-0.040	0.191
Arg596	-0.291	-8.592	7.243	-1.640
Ser597	-0.050	0.391	-0.070	0.272
Cys598	-0.050	0.171	-0.039	0.082
Trp599	-1.374	-0.004	-0.046	-1.424
His600	-1.178	0.875	-0.019	-0.323
Leu601	-0.071	0.364	-0.110	0.182
Gly602	-0.067	0.170	-0.102	0.000
Ala603	-0.828	0.409	-0.197	-0.616
Val604	-0.254	0.102	-0.125	-0.276

trp605	-0.034	0.238	-0.138	0.066
Ala606	-0.056	-0.260	-0.104	-0.419
Leu607	-0.940	-0.765	0.032	-1.672
Ser608	-0.025	-0.072	-0.026	-0.123
Val671	-0.059	-0.340	0.085	-0.315
Ala672	-0.071	0.035	-0.073	-0.109
Ile673	-0.453	15.345	-9.761	5.131
Fe^{+2}674	-0.048	-42.645	20.330	-22.363

Table S5. Per-residue energy decomposition complex 3O8Y and compound **11**.

	Energy Decomposition Analysis (kcal/mol)			
Residue	van der Waals Avg.	Electrostatic Avg.	Polar Solvation Avg.	TOTAL Avg.
Trp147	-0.028	0.018	0.007	-0.004
phe151	-0.031	0.066	-0.001	0.035
Ser154	-0.014	0.030	0.000	0.016
Ile155	-0.008	-0.020	0.005	-0.023
Phe169	-0.017	-0.007	0.002	-0.022
Val175	-0.065	0.021	-0.020	-0.063
Phe177	-0.989	0.020	0.008	-0.960
Val178	-0.081	0.026	-0.039	-0.093
Asn180	-0.104	-0.267	0.155	-0.216
Tyr181	-2.043	-2.068	0.641	-3.470
Ser182	-0.018	-0.009	0.020	-0.007
Ala184	-0.019	-0.028	0.045	-0.001
Met185	-0.056	-0.008	0.018	-0.046
Met194	-0.027	-0.020	-0.013	-0.060
Phe197	-0.071	-0.001	0.002	-0.070
Asn295	-0.013	-0.064	0.020	-0.058
Gln303	-0.032	-0.018	-0.023	-0.072
Phe359	-0.252	-0.216	0.087	-0.381
his360	-0.060	-0.071	0.007	-0.124
Val361	-0.023	-0.147	0.030	-0.140
His362	-0.232	-0.505	0.101	-0.636
Gln363	-1.553	-6.476	1.820	-6.209
Thr364	-0.147	-0.055	0.016	-0.186
Ile365	-0.054	-0.127	-0.003	-0.185
Thr366	-0.055	0.017	-0.002	-0.040
His367	-1.802	0.427	-0.128	-1.503
leu368	-1.334	0.194	-0.046	-1.186
Leu369	-0.083	0.017	-0.022	-0.089
Arg370	-0.027	0.661	-0.282	0.351
Thr371	-0.080	0.074	-0.015	-0.021
His372	-1.850	0.447	-0.229	-1.633
Leu373	-0.434	0.193	-0.014	-0.256
Val374	-0.018	0.053	-0.007	0.028
Ser375	-0.016	0.036	-0.003	0.017
Glu376	-0.082	-0.050	0.024	-0.107
Phe402	-0.015	0.016	0.004	0.006
Thr403	-0.051	0.096	0.000	0.045
Ile404	-0.031	0.076	-0.002	0.042
Ala405	-0.025	0.048	-0.007	0.016
Ile406	-0.494	0.071	-0.023	-0.445
Asn407	-1.286	0.104	0.002	-1.180
Thr408	-0.099	0.032	-0.006	-0.073
Lys409	-0.091	-0.337	0.232	-0.196
Ala410	-0.730	-0.215	-0.020	-0.965
Arg411	-0.694	0.386	-0.033	-0.341
Glu412	-0.030	0.250	-0.214	0.006
Gln413	-0.064	-0.068	0.016	-0.116
Leu414	-1.913	-0.024	0.033	-1.904
Ile416	-1.357	-0.037	0.004	-1.390
Cys416	-0.039	0.057	-0.005	0.013
Gly419	-0.061	0.005	-0.005	-0.061
Leu420	-1.292	0.336	-0.024	-0.980
Phe421	-2.066	-0.179	0.004	-2.241
Asp422	-0.108	0.476	-0.105	0.263

Lys423	-0.384	-0.204	0.184	-0.405
Ala424	-1.077	-0.933	0.040	-1.970
Asn425	-0.920	-1.604	0.111	-2.412
Ala426	-0.025	0.036	0.002	0.012
Thr427	-0.028	0.011	-0.006	-0.023
Gly428	-0.017	-0.100	0.030	-0.087
His432	-0.077	-0.356	0.065	-0.368
Val433	-0.008	-0.050	0.011	-0.047
Met435	-0.012	0.019	0.006	0.013
Val436	-0.023	0.034	0.001	0.011
His550	-0.093	0.149	-0.076	-0.019
Val553	-0.057	-0.027	-0.016	-0.099
Asn554	-0.180	0.194	-0.080	-0.067
Gln557	-0.082	0.059	0.008	-0.015
Tyr558	-0.026	-0.012	0.007	-0.031
Ala561	-0.021	0.018	-0.009	-0.012
Ser562	-0.009	-0.015	0.008	-0.016
Ile564	-0.026	-0.026	0.015	-0.038
Ala567	-0.013	0.023	-0.020	-0.010
Pro568	-0.032	-0.016	0.036	-0.012
pro569	-0.408	0.043	0.051	-0.313
Thr570	-0.090	-0.036	0.021	-0.104
Pro592	-0.042	0.029	0.033	0.020
Gly595	-0.074	0.088	-0.034	-0.020
Arg596	-0.928	0.377	0.580	0.029
Ser597	-0.111	0.085	-0.049	-0.076
Cis598	-0.083	0.066	-0.021	-0.037
Trp599	-1.616	-0.137	0.041	-1.712
His600	-1.343	-3.136	0.419	-4.060
Leu601	-0.108	0.106	-0.033	-0.035
Gly602	-0.099	-0.035	0.003	-0.132
Ala603	-1.221	-0.341	0.053	-1.508
Val604	-0.459	-0.203	0.005	-0.658
Trp605	-0.083	0.027	0.013	-0.043
Ala606	-0.382	0.175	0.091	-0.116
Leu607	-1.747	0.009	0.077	-1.660
Ser608	-0.063	-0.025	0.044	-0.043
Val671	-0.074	0.016	0.001	-0.057
Ala572	-0.045	-0.011	0.002	-0.055
Ile673	-0.881	-0.847	0.976	-0.751
Fe^{+2}674	-0.043	-2.670	1.138	-1.575

i
